# Glucose sensing in the pancreatic beta cell: a computational systems analysis

**DOI:** 10.1186/1742-4682-7-15

**Published:** 2010-05-24

**Authors:** Leonid E Fridlyand, Louis H Philipson

**Affiliations:** 1 Department of Medicine, The University of Chicago, Chicago, IL, USA 60637

## Abstract

**Background:**

Pancreatic beta-cells respond to rising blood glucose by increasing oxidative metabolism, leading to an increased ATP/ADP ratio in the cytoplasm. This leads to a closure of K_ATP _channels, depolarization of the plasma membrane, influx of calcium and the eventual secretion of insulin. Such mechanism suggests that beta-cell metabolism should have a functional regulation specific to secretion, as opposed to coupling to contraction. The goal of this work is to uncover contributions of the cytoplasmic and mitochondrial processes in this secretory coupling mechanism using mathematical modeling in a systems biology approach.

**Methods:**

We describe a mathematical model of beta-cell sensitivity to glucose. The cytoplasmic part of the model includes equations describing glucokinase, glycolysis, pyruvate reduction, NADH and ATP production and consumption. The mitochondrial part begins with production of NADH, which is regulated by pyruvate dehydrogenase. NADH is used in the electron transport chain to establish a proton motive force, driving the F_1_F_0 _ATPase. Redox shuttles and mitochondrial Ca^2+ ^handling were also modeled.

**Results:**

The model correctly predicts changes in the ATP/ADP ratio, Ca^2+ ^and other metabolic parameters in response to changes in substrate delivery at steady-state and during cytoplasmic Ca^2+ ^oscillations. Our analysis of the model simulations suggests that the mitochondrial membrane potential should be relatively lower in beta cells compared with other cell types to permit precise mitochondrial regulation of the cytoplasmic ATP/ADP ratio. This key difference may follow from a relative reduction in respiratory activity. The model demonstrates how activity of lactate dehydrogenase, uncoupling proteins and the redox shuttles can regulate beta-cell function in concert; that independent oscillations of cytoplasmic Ca^2+ ^can lead to slow coupled metabolic oscillations; and that the relatively low production rate of reactive oxygen species in beta-cells under physiological conditions is a consequence of the relatively decreased mitochondrial membrane potential.

**Conclusion:**

This comprehensive model predicts a special role for mitochondrial control mechanisms in insulin secretion and ROS generation in the beta cell. The model can be used for testing and generating control hypotheses and will help to provide a more complete understanding of beta-cell glucose-sensing central to the physiology and pathology of pancreatic *β*-cells.

## Background

**Figure 1:**
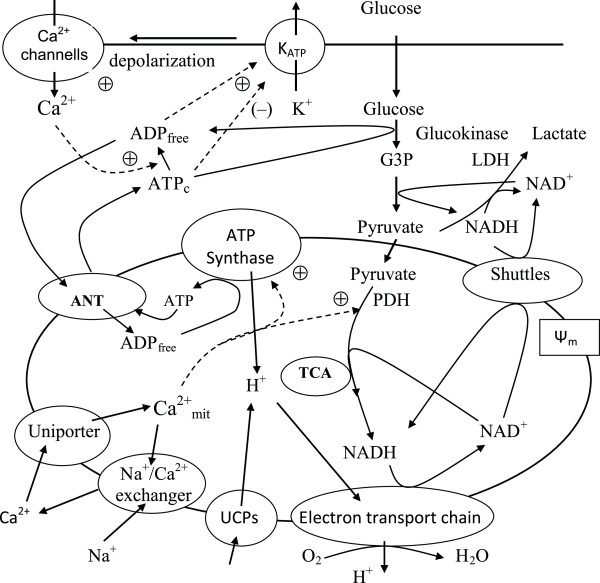
**Schematic diagram of biochemical pathways involved in energy metabolism and Ca^2+ ^handling in the pancreatic *β*-cell**. Glucose equilibrates across the plasma membrane and is phosphorylated by glucokinase to glucose 6-phosphate, which initiates glycolysis. Lactate dehydrogenase (LDH) converts a portion of pyruvate to lactate. Pyruvate produced by glycolysis preferentially enters the mitochondria and is metabolized in the tricarboxylic acid (TCA) cycle, which then yields reducing equivalents in the form of NADH and FADH2. The transfer of electrons from these reducing equivalents through the mitochondrial electron transport chain is coupled with the pumping of protons from the mitochondrial matrix to the intermembrane space. The resulting transmembrane electrochemical gradient drives the ATP synthesis at ATP-synthase. Part of the protons may leak back through uncoupling proteins (UCPs). The shuttle systems are required for the transfer of reducing equivalents from the cytoplasm to the mitochondrial matrix. Calcium handling proteins such as the uniporter and Na^+^/Ca^2+ ^exchanger regulate Ca^2+ ^handling in mitochondria. ATP is transferred to the cytosol, raising the ATP/ADP ratio. This results in the closure of the ATP sensitive K^+ ^channels (katp), which in turn leads to depolarization of the cell membrane. In response, the voltage-sensitive Ca^2+ ^channels open, promoting calcium entry and increasing the cytoplasmic Ca^2+^. ATP_c _and ADP_free _are the free cytosolic form of ATP and ADP, G3P is the glyceraldehydes 3-phosphate, PDH is the pyruvate dehydrogenase, ANT is the adenine nucleotide translocase, Ψ_m _is the mitochondrial membrane potential. Solid lines indicate flux of substrates, and dashed lines indicate regulating effects, where (+) represents activation and (-) repression.

The appropriate secretion of insulin from pancreatic *β*-cells is critically important for energy homeostasis. Pancreatic *β*-cells are adapted to sense blood glucose and other secretagogues to adjust insulin secretion according to the needs of the organism. Rather than activating specific receptor molecules, glucose is metabolized to generate downstream signals that stimulate insulin secretion. Pancreatic *β*-cells respond to rising blood glucose by increasing oxidative metabolism, leading to increased ATP production in mitochondria and in an enhanced ratio of ATP to ADP (ATP/ADP) in the cytoplasm [1-3]. The increase in intracellular ATP/ADP closes the ATP-sensitive K^+ ^channels (K_ATP_), decreasing the hyperpolarizing outward K^+ ^flux. This results in depolarization of the plasma membrane, influx of extracellular Ca^2+ ^through the voltage-gated Ca^2+ ^channels, a sharp increase in intracellular Ca^2+ ^and activation of protein motors and kinases, which then mediate exocytosis of insulin-containing vesicles [2-5]. The currently accepted processes of glucose metabolism and Ca^2+ ^handling in the cytoplasm and mitochondria of *β*-cells considered in this analysis are summarized in Figure 1[1-4].

A brief summary of these processes includes the following steps. Glucose enters *β*-cells by facilitated diffusion through glucose transporters (GLUT1 and 2). While this process is not limiting in *β*-cells [6], the next irreversible step, glucose phosphorylation, is catalyzed by a single enzyme, glucokinase (GK). This enzyme is specific for metabolic control in the *β*-cell and hepatocyte, because the K_m _of GK for glucose is ~8 mM, a value that is almost two orders of magnitude higher than that of any other hexokinase. This step appears to be rate limiting for *β*-cell glycolytic flux under normal physiological conditions, so that GK is regarded as the *β*-cell 'glucose sensor' [1,3], underlying the dependence of the *β*-cell insulin secretory response to glucose in the physiological range.

Pyruvate is the main end product of glycolysis in *β*-cells and essential for mitochondrial ATP synthesis. In the mitochondrial matrix, pyruvate is oxidized by pyruvate dehydrogenase to form acetyl-coenzyme A (acetyl-CoA). Acetyl-CoA enters the tricarboxylic acid (TCA) cycle to undergo additional oxidation steps generating CO_2 _and the reducing equivalents, flavin adenine dinucleotide (FADH2) and NADH. Oxidation of reducing equivalents by the respiratory chain is coupled to the extrusion of protons from the matrix to the outside of the mitochondria, thereby establishing the electrochemical gradient across the inner mitochondrial membrane (Figure 1). The final electron acceptor of these reactions is molecular oxygen, as in other eukaryotic cells. The electrochemical gradient then drives ATP synthesis at the F_1_F_0_-ATPase complex to phosphorylate mitochondrial ADP, thereby linking respiration to the synthesis of ATP from ADP and inorganic phosphate (Figure 1). Adenine nucleotide translocase (ANT) exchanges matrix ATP for ADP to provide ATP for energy consuming processes in the cytosol. Some cytosolic ATP is also produced in the latter part of glycolysis. However, this appears to be of minor consequence relative to that subsequently generated in the mitochondria, which represents an estimated 90% of the total *β*-cell ATP production [7,8].

The cytoplasmic Ca^2+ ^signal is coupled to mitochondrial Ca^2+ ^handling (Figure 1). The balance of Ca^2+ ^influx and efflux determines the matrix Ca^2+ ^level involving the Ca^2+ ^uniporter and the mitochondrial Na^+^/Ca^2+ ^exchanger, respectively. Ca^2+ ^influx into mitochondria is amplified by hyperpolarization of the inner mitochondrial membrane [9,10]. Inside the organelle, Ca^2+ ^activates several matrix dehydrogenases (for example, pyruvate dehydrogenase). Mitochondrial Ca^2+ ^may also directly stimulate ATP synthase [11]. The nutrient-dependent Ca^2+ ^rise in the cytosol further activates ATP hydrolysis [7,10,12,13].

An important *β*-cell specialization is the very low expression of lactate dehydrogenase (LDH), the enzyme catalyzing the conversion of pyruvate to lactate [1,14,15]. A low level of LDH expression in insulin-secreting cells is important to preferentially channel pyruvate towards mitochondrial metabolism (see [1,10,16]). However, the low LDH levels likely leads to activation of compensatory mechanisms because NAD^+^-dependent glycolytic enzymes (e.g., glyceraldehyde 3-phosphate dehydrogenase) require that cytoplasmic NADH must be re-oxidized to NAD^+^. This reaction is usually catalyzed by LDH, but because *β*-cells cannot use this pathway effectively, these cells must re-oxidize cytoplasmic NADH by activation of two mitochondrial hydrogen shuttles (Figure 1), the malate-aspartate shuttle and the glycerol phosphate shuttle [15,17-19].

Glucose signaling in *β*-cells has several other peculiarities, including generation of multiple oscillations in metabolism, mitochondrial membrane potential Ψ_m_) and NADH, mitochondrial and cytoplasmic Ca^2+ ^and, ultimately, the oscillations of insulin secretion [5,20-23]. The coupling of these various oscillators is not clearly understood. In addition, the respiratory rate is lower and relative leak activity is higher in isolated *β*-cell mitochondria (as found in a cultured *β*-cell line) compared with isolated mitochondria from skeletal muscle [24,25]. These observations need clarification to better understand how mitochondrial processes are linked with insulin secretion.

The unique character of the *β*-cell response to glucose is usually attributed solely to glucokinase. Because of its near-dominant control of glycolytic flux, this enzyme is thought to govern the ATP/ADP ratio and insulin secretion almost exclusively [1,3]. While glucokinase certainly exerts a critical level of control on downstream events, other cytoplasmic and mitochondrial processes also play an essential role in glucose-stimulated insulin secretion (GSIS) [1,2,10]. In particular the relatively high flexibility of the ATP/ADP ratio in *β*-cells may be accounted for, at least partly, by mitochondrial peculiarities as well as by properties of glucokinase [24,26,27]. For these reasons it is critical to develop a comprehensive understanding as to how cytoplasmic and intramitochondrial fuel metabolism is coupled to fuel availability and thereby "sensed."

The goal of this work is to determine the contribution of the cytoplasmic and mitochondrial processes regulating GSIS using a mathematical modeling approach. Mathematical modeling can be a powerful systems biology tool allowing quantitative descriptions of the control individual components exert over the whole biological system. Several mathematical approaches in the literature have provided quantitative estimates of energetic and mitochondrial processes in pancreatic *β*-cells. However, these models are limited in the pathways that are considered, so that a more comprehensive approach is now necessary.

The first detailed *β*-cell model was developed by Magnus and Keizer [28-30]. However, several mechanisms used for simulations in this model have recently been reevaluated. For example, steady-state electron transport and the F_1_F_0 _ATPase proton pump were modeled according to the "six states proton pump mechanism" [28]. This mechanism does not correspond to the present understanding of the function of the electron transport chain (ETC) and the mitochondrial F_1_F_0 _adenosine trisphosphatase (see for example [31]). Models of the LDH and NADH shuttles were not included, and mitochondrial fluxes may also have been overestimated in this model (see below). The main goal of these models was to examine the possible mechanisms underlying oscillations in pancreatic *β*-cells, not biochemical regulation of *β*-cell glucose sensitivity that we are focused on here.

A complex kinetic model of the metabolic processes in pancreatic *β*-cells based on *in vitro *enzyme kinetics was recently developed [32]. However, while heroically complicated models with numerous parameters and enzyme activities are interesting, they require data on *in vivo *enzyme activities and coefficients that are not readily available.

Enzyme activity measurements *in vitro*, often used in models, may not reflect enzyme activity *in vivo *[33]. For example, experimental kinetic data for isolated mitochondria and the parameters evaluated for mitochondrial processes from experiments with intact cells may differ significantly [34]. For these and other reasons previous models of pancreatic *β*-cell energetics and mitochondrial calcium regulation fall short of a comprehensive explanation of the mechanisms of *β*-cell sensitivity.

To address this we have developed a specific quantitative, kinetic model (see Appendix) of the core processes of *β*-cell cytoplasmic and mitochondrial energetic based on a simplified map of the biochemical pathways schematized in Figure 1. We included the most recent experimental characterizations of the majority of processes in the model to insure accuracy. However, for simplification, we modeled only those regulatory couplings that we have deemed most crucial for the *β*-cell metabolic regulation based on experimental evidence. The model includes the dynamic equations for cytoplasmic ADP, NADH and glyceraldehyde 3-phosphate, mitochondrial Ψ_m _and NADH, mitochondrial and cytoplasmic Ca^2+ ^and pyruvate. When available we used the values of the coefficients determined for living cells rather than for isolated enzymes and cell-free mitochondria (Appendix).

We show that this model has qualitative properties consistent with expectations for the pancreatic *β*-cell including showing appropriate oscillations in mitochondrial metabolism and Ca^2+ ^concentration. The model also reproduces simultaneous measurements of the behavior of multiple constituents within the cytoplasm and mitochondria such as NADH, Ca^2+ ^and Ψ_m _at high temporal resolution. We also discuss specific differences in muscle and *β*-cell mitochondrial function, providing insight into essential control properties of the *β*-cell. Furthermore, predictions on the dynamics of as yet unmeasured molecules could be made, and the model further tested by verifying these predictions.

Nutrient-stimulated insulin secretion in *β*-cells is impaired in the diabetic state. This may result from impaired glucose-induced ATP/ADP ratio elevation in *β*-cells [26,35]. Furthermore, it is becoming increasingly clear that the development of type 2 diabetes is associated with mitochondrial dysfunction [27,35-37]. Insulin signaling also effects mitochondrial function in *β*-cells [38]. Thus, knowledge of the mechanisms of regulation of ATP production and consumption are central to understand *β*-cell glucose-sensing and mechanisms of dysfunction in type 2 diabetes.

## Results and Discussion

### Steady state stimulation with a step increase in glucose concentration

**Figure 2:**
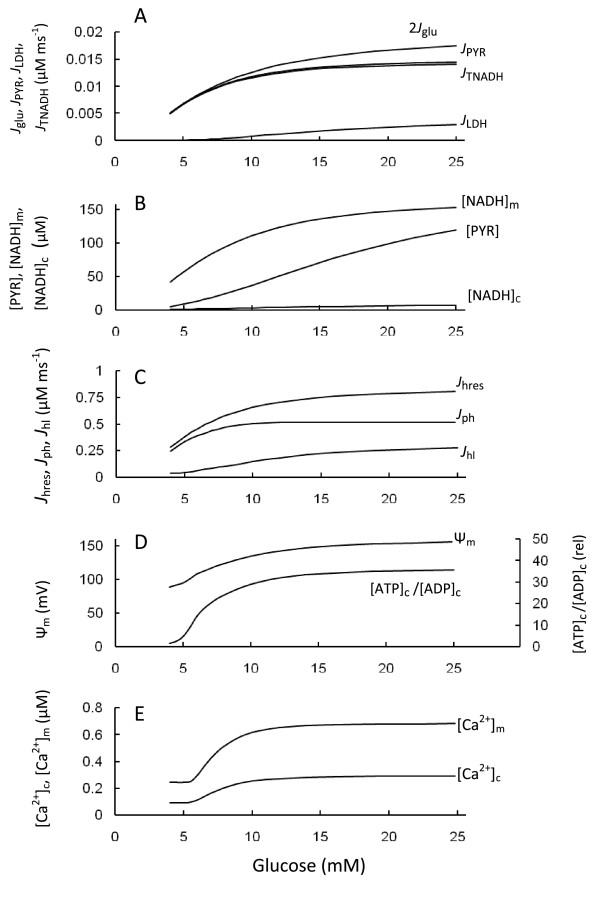
**Effect of increasing glucose on cell energetics**. Extracellular glucose concentration was varied and the steady-state simulations of the model parameters represented. Simulations were run with the basic set of parameters (Tables 2 and 3). A: *J*_glu _is the rate of the glucokinase reaction (for comparison with other fluxes the amount of 2 *J*_glu _is represented since two pyruvate molecules are synthesized from one molecule of glucose), jpyr is the rate of pyruvate decarboxylation, jtnadh is the flux through the NADH shuttles measured as the rate of cytoplasmic NAD^+ ^production from cytoplasmic NADH, *J*_LDH _is the lactate flux catalyzed by lactate dehydrogenase; B: [NADH]_c _and [NADH]_m _are cytoplasmic and mitochondrial NADH, [Pyr] is the pyruvate concentration, [G3P] is the cytoplasmic glyceraldehydes 3-phosphate concentration; C: Jhres is the rate of proton pumping through ETC, *J*_ph _is the proton flux through the F_1_F_0 _ATPase, Jhi is the leak of protons from mitochondria; D: Ψ_m _is the mitochondrial membrane potential, [ATP]_c_/[ADP]_c _is the cytoplasmic ATP/ADP ratio; E: [Ca^2+^]_c _and [Ca^2+^]_m _are the concentration of free Ca^2+ ^in cytoplasm and mitochondria.

The model was used to simulate data obtained using several experimental protocols. Under resting low glucose concentration the simulated values of the cytoplasmic and mitochondrial variables are consistent with experimentally reported data as indicated in Table 1. Then the model was used to examine the steady-state changes of the state variables and fluxes. Figure 2 shows the responses of model simulation to steps in the glucose concentration observed in successive steady-states. Glucokinase catalyzed the rate-limiting step of glycolysis with a steep dependence on glucose concentration in the range 4-25 mM. Enhancement of glucose concentration led to an increase in glycolytic flux, glyceraldehyde 3-phosphate (G3P) and pyruvate concentrations (Figure 2A,B). This process accelerated pyruvate reduction and decarboxylation leading to increased [NADH]_m _(Figure 2B). [NADH]_m _was oxidized by the ETC, raising the rate of mitochondrial O_2 _consumption. Oxidation of mitochondrial NADH by the respiratory chain increased the membrane potential directly via protons pumped out of the matrix. Ψ_m _was dissipated by proton-leak reactions and the activity of the phosphorylation apparatus, which included the phosphate carrier and the ATP synthase (Figure 1). The net result of these processes was establishment of an elevated Ψ_m_. The hyperpolarization of the inner mitochondrial membrane resulted in increased ATP production by F_1_F_0 _ATPase, decreased [ADP]_c _and a corresponding increased ATP/ADP ratio (Figure 2D). The phosphorylation rate (*J*_ph_) reached saturation at high glucose concentration (Figure 2C) as a consequence of decreased [ADP]c and saturated Ψ_m_(see Equation 7 and Figure 12 in Appendix). Simultaneously, [Ca^2+^]_c _increased with increased ATP/ADP ratio according to the empirical Equation 23 (Appendix). We also simulated the steady-state response of free mitochondrial matrix Ca^2+ ^to changes in cytoplasmic Ca^2+ ^concentration, Ψ_m _and finally glucose (Figure 2E).

**Table 1:** Stimulated steady-state values for low glucose (5 mM) (see text for explanations)

Parameters	Simulated value	Experimental value	References
[Ca^2+^]_c_	0.09 *μ*M	~0.1 *μ*M	[49,52]
[G3P]	2.79 *μ*M		
[PYR]	8.62 *μ*M		
[ATP]_c_/[ADP]_c_	4.6	~3 - 6	[7,12,51]
[NADH]_c_	0.97 *μ*M		
Ψ_m_	94.7 mV		
[NADH]_m_	57.2 *μ*M	60 *μ*M	[47]
[Ca^2+^]_m_	0.242 *μ*M	0.25 *μ*M	[48]

As expected, our simulations were consistent with experimental data. Glucose utilization increased lactate synthesis, O_2 _consumption and CO_2 _production [1,7,39,40]. Both cellular [G3P] and [PYR] increased after simulating increased extracellular glucose (Figure 2B). This result is consistent with the finding of increased glycolytic intermediates and pyruvate after glucose challenge in the INS1 *β*-cell line [41] as well as the increase in Ψ_m _with increased glucose in mouse islets [20-22,42-44].

An accurate measurement of lactate output in *β*-cells from isolated islets is difficult to obtain because LDH expression in non-*β*-cells is considerably higher than in *β*-cells, and high rates of lactate output may also originate from cells in the centers of isolated islets that are prone to oxygen depletion and necrosis [39,45]. However, the oxidative production of CO_2 _from [3,4-^14^C]glucose represented close to 100% of the total glucose utilization in purified rat *β*-cells [39] indicating that lactate output should not exceed several percent. Very low lactate output was also found in *β*-cell lines [46]. Our simulated small lactate output in Figure 2A is consistent with these experimental data.

The results of the simulation (Table 1 and Figure 2B) were also consistent with the range of measured [NADH]_m _reported previously. For example, the concentration of free NADH in mitochondria of intact pancreatic islets at resting glucose levels (4-5 mM) is about 60 *μ*M and the maximum mitochondrial glucose-induced increase in free NAD(P)H reached 75 *μ*M [47]. The simulated increased [Ca^2+^]_c _versus glucose concentration (Figure 2E) was also in agreement with previous reports (see for example [42,48-50]).

Several studies have confirmed an increase in the ATP/ADP ratio in response to high glucose (see for e.g. [3,7,12,26,51]. A simultaneous rise in ATP/ADP and NADH/(NAD^+ ^+ NADH) ratio was found in rat islets [52], and NAD^+^/NADH was increased in rat *β*-cells and in the MIN6 *β*-cell line in response to high glucose [53]. The rise in ATP/ADP ratio as well as in relative NAD(P)H, Ψ_m_, [Ca^2+^]_m _and oxygen consumption were also observed with glucose stimulation in control INS-1 cells [54,55]. Our simulations are generally consistent with these data.

### Regulation at the mitochondrial level

The model suggests a possible reconciliation of several apparent contradictions between live cell experimental data and regulation of mitochondrial energetics obtained in experiments with isolated mitochondria.

1. A basic principle of mitochondrial energetics is given by the inverse relationship between the respiratory flux and Ψ_m_, i.e. the higher Ψ_m_, the lower the respiration rate [24,56]. We simulated this relationship using Equation 5C (Appendix). However, the electron transport rate (*J*_hres_) and O_2 _consumption increased simultaneously with Ψ_m _in our simulation of the *β*-cell (see Figure 2C) as well as *in vivo *(see above). The model offers the following explanation of this contradiction. In our model (as in living cells) the electron transport rate (Equation 5) depends on at least two factors: one is a decrease in the electron transport rate with an increase in Ψ_m _(Equation 5C) but another factor is an increase of this rate with increased substrate concentration (NADH) (Equation 5A). Increasing the electron transport rate simultaneously with Ψ_m _means that the enhancement of *J*_hres _as a result of the increased [NAPH]_m _was greater than its decrease with the rise of Ψ_m _following the step increase in glucose. Substrate concentrations are usually maintained at constant or saturated levels in experiments with isolated mitochondria, where one can only see inhibition of the electron transport rate with increased Ψ_m_.

2. The respiratory control hypothesis for ATP production in intracellular mitochondria was based on experiments with isolated mitochondria which found that ADP availability to the ATP-synthase is the limiting factor for mitochondrial ATP production [57], that is, the rate of ATP synthesis should decrease with decreased [ADP]_c_. This mechanism corresponds to Equation 7A in our model. Experimentally this hypotheses has been tested in permeabilized clonal *β*-cells, where ATP/ADP ratios can be externally fixed showing that a decrease in [ADP] led to decreased O_2 _consumption [3]. However, an increased ATP/ADP ratio (usually due to decreased [ADP]_c_) coincidentally with increased respiration rate and oxidative phosphorylation has been firmly established for pancreatic *β*-cells as a signal for GSIS in response to increased glucose [1,3,7,12,26,51,55]. Similar results were obtained in our simulation of *β*-cell shown in Figure 2D. At first glance these data seem inconsistent with the expected inhibition of respiration with decreased ADP concentration [3,55].

Our analysis resolves this apparent contradiction. In our model the ATP synthesis rate is dependent on at least two factors: one is a decreased ATP synthesis rate with decreased [ADP]_c _(Equation 7A) but another factor is an increased ATP synthesis rate with increased Ψ_m_(Equation 7B). Our simulation shows that enhancement of ATP production with increasing Ψ_m _was greater than its decrease as a result of decreasing [ADP]_c _following a step increase in glucose. As a result, ATP synthesis and respiration rate increase despite decreased [ADP]_c _and the ATP/ADP ratio increased with a step glucose increase (Figure 2D). These simulations imply that glucose challenge can lead to simultaneous increases in Ψ_m_, the ATP/ADP ratio and in the rates of mitochondrial ATP synthesis and respiration.

The concentrations of free ATP and ADP in the cytoplasm were used in our model since only free molecules can take part in reactions. However, the free ATP concentration is close to its total concentrations, whereas the fraction of bound ADP may be substantial [58,59]. On the other hand, most estimated ATP/ADP ratios are based on measurements of total nucleotide content [7,12,51]. For this reason, the measured ATP/ADP ratio of total ATP and ADP nucleotide content is likely to be substantially smaller than ratio of concentration of the free components, simply because the measured total ADP content includes bound ADP. Therefore, it is not surprising that the simulated ATP/ADP ratio change in Figure 2D using free nucleotide concentrations is greater than that in published experimental data (see for example [7,12,51].

According to our simulation only a small increase in the ATP concentration occurred following glucose challenge (not shown). A decrease in the free [ADP]_c _is the main factor leading to an increase in the ATP/ADP ratio following increased glucose in Figure 2D. This simulation is in agreement with experimental data and can be a consequence of the initial high ATP/ADP ratio even with a low glucose level in our model (see Table 1). For this reason, the ATP concentration cannot be increased significantly if the total adenine nucleotide concentration is kept constant, whereas the relative [ADP] may undergo a pronounced decrease (see our previous publication [26] for a detailed consideration of this question).

### Decreased Ψ_m _and respiratory activity regulate mitochondrial glucose sensitivity in *β*-cells

*β*-cell regulatory mechanisms endow this cell type with unique metabolic properties to control insulin secretion in comparison with metabolism in other cell types. For example, liver cells maintain a stable ATP/ADP equilibrium while respiring at widely varying rates [60]. Cardiac myocytes can increase, by three- to sixfold, the rate of cardiac power generation, myocardial oxygen consumption, and ATP turnover in the transition from rest to intense exercise [61]. Nevertheless, at high work states the myocardial ATP and ADP concentrations are maintained at a relatively constant level despite the increased turnover rates [34,62].

**Figure 3:**
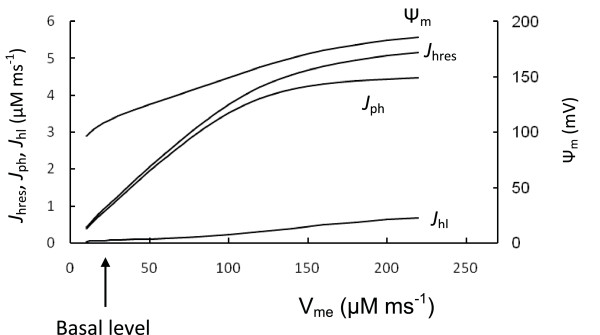
**Comparison with muscle cell mitochondria**. To compare *β*-cell and muscle cell mitochondria function we increased step by step the maximal rate of respiration (V_me_, Equation 5) from basal *β*-cell level (arrow) (Table 3) and calculated the respiration rate (as J_hres_), phosphorylation rate (as *J*_ph_), leak (J_hl_) and Ψ_m _in response to the maximal rate of proton pumping through the ETC (V_me_). Coefficients for F_1_F_0 _ATPase activity were unchanged. [NADP]_m _(1570 *μ*M) and ADP (700 *μ*M) were set to be constant at high levels. Note the rate of ATP production (represented as *J*_ph_) increased significantly as well as Ψ_m_with an increase of the maximal rate of respiration (compare with Figure 2). All other parameters were set as in Figure 2.

Specific *β*-cell respiratory mechanisms can be illustrated by comparing isolated mitochondria from skeletal muscle and cultured *β*-cells. The rate of respiration was higher (>5.5 fold) and the relative leak rate was significantly lower at any Ψ_m _value in isolated mitochondria from skeletal muscle than in those from cultured *β*-cells [24,25]. We examined how these differences effect mitochondrial function by simulating the conditions of work in muscle mitochondria (Figure 3). Mitochondrial NADH and cytoplasmic ADP concentration are maintained at a relatively high and constant level in muscle cells [34,62]. To simulate this, the concentration of [NADH]_m _was set as a constant reflecting this concentration for high glucose level in a *β*-cell (25 mM). [ADP]_c _was also set to an elevated constant level (700 mM), that was 5-fold higher than the calculated [ADP]_c _level (at 9 mM glucose) in *β*-cells.

Figure 3 shows the results of simulations in which the maximal rate of ETC (V_me_) was increased in steps. Mitochondrial F_1_F_0 _ATPase activity (V_mph_) was unchanged. Simulated Ψ_m _and the rate of ATP production (*J*_ph_) were significantly increased with an increased V_me_, such that F_1_F_0 _ATPases work with maximal activity under these conditions (compare *J*_ph _in Figure 2C and Figure 3). This can be explained by the high Ψ_m _(more electronegative) as well as by the increased [ADP]_c _in simulated muscle cells in comparison with *β*-cells. Note that the rate of ATP production (*J*_ph_) depended only slightly on Ψ_m _change when Ψ_m _was increased above 160 mV, since these levels of Ψ_m _were saturating for F_1_F_0 _ATPase activity (Appendix, Figure 12).

This indicates that the F_1_F_0 _ATPase can work in muscle cells with maximal productivity during increased respiration activity because ADP concentration and Ψ_m _are supported at relatively higher levels. It thus appears that a decrease in the efficiency of mitochondrial energy production with decreased Ψ_m _can lead to a relatively high degree of control on the phosphorylation potential in *β*-cells, i.e. a change in Ψ_m _leads to a large change in *J*_ph_. Interestingly, the simulated relative leak (*J*_h1_) magnitude was significantly lower in the muscle cell simulation in comparison with respiration rate (evaluated as *J*_hres_) at increased V_me _even with invariant coefficients for the proton leak, since the rates of respiration and ATP production were highly increased but a coefficient of leak (*J*_h1_) would remain as constant (Figure 3).

Our simulations help explain the data of Affourit and Brand [24,25] showing decreased respiratory and increased relative leak activity in isolated *β*-cell mitochondria. This suggests that mitochondrial glucose sensitivity in *β*-cells results from decreased respiratory activity compared with F_1_F_0 _ATPase activity. This leads to mitochondrial work at decreased Ψ_m _that is in the region where variations in Ψ_m _should result in an increased sensitivity to glucose. Decreased respiratory activity in *β*-cells leads to a decreased ATP production rate by the F_1_F_0 _ATPase. However, this gives *β*-cells the ability to adaptively change the ATP/ADP ratio in response to changes in glucose concentration.

Interestingly, the oxidative phosphorylation rate (per g dry weight) was significantly lower in pancreatic islets even in high glucose, compared with brain or heart [7], supporting our suggestion regarding decreased respiratory activity in *β*-cells.

### Changes in leak activity and the role of uncoupling agents

**Figure 4:**
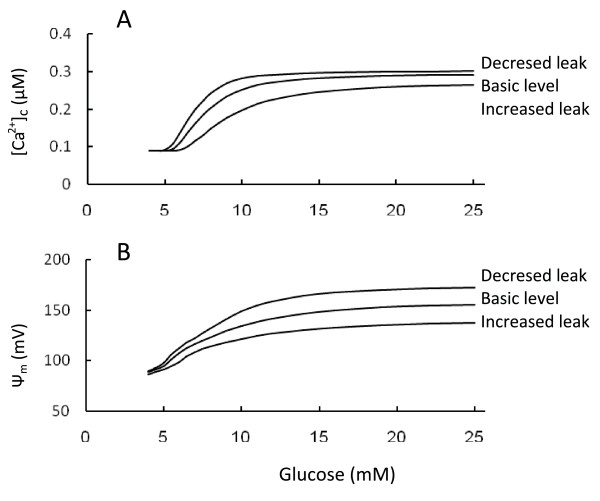
**Effect of increasing glucose at different leak activity**. A. [Ca^2+^]_c_; B. Ψ_m_. To simulate the increase leak activity we magnified the proton leak rate twofold by increasing P_lr _(P_lr _= 0.0036 *μ*M ms^-1^) in Equation 8. As expected, one effect was to reduce the inner membrane potential, and thus the ATP/ADP ratio. To simulate the decreased leak activity we diminished the proton leak rate twofold by decreasing P_ir _in Equation 8 (P_ir _= 0.0 *μ*M ms"^1^) (see text for explanation). All other parameters were set as in Tables 2 and 3.

Our model features increasing proton-leak with increased Ψ_m _(Equation 8, Appendix). We simulated how changes in leak activity affect the response of the variables. To do this we increased the regulated coefficient of proton leak (P_1r _in Equation 8, Appendix) threefold leading to an increase in the total proton leak rate by twofold (Figure 4). As expected, one effect was to reduce the inner membrane potential that causes a corresponding right-shift in the ATP/ADP ratio and [Ca^2+^]_c _response to glucose. To simulate decreased leak activity (for example following decreased uncoupling protein expression) we set the regulated proton leak coefficient equal to zero in Equation 8 (P_1r _= 0) (Figure 4). In this way the general leak activity was decreased by 50%. Decreased leak activity increased Ψ_m _and reduced the sensitivity range of the inner membrane potential to glucose leading to a left-shift in the ATP/ADP ratio and [Ca^2+^]_c _response.

These simulations show that proton leak can modulate GSIS by shifting the dependence on glucose of the ATP/ADP ratio and [Ca^2+^]_c_, altering cellular sensitivity to glucose challenge. This effect of proton leak is only possible when the ATP/ADP ratio can be regulated by changes in Ψ_m_, i.e. when Ψ_m _lies below the *β*-cell maximal level for ATP production. On the other hand, in muscle cells Ψ_m _can be maintained at a high level (see above) and changes in Ψ_m _exert an insignificant effect on the ATP production rate.

This role of uncoupling agents can be illustrated by considering the experimental data for the principal *β*-cell uncoupling protein 2 (UCP2) (see for review [63,64]. For example, overexpression of UCP2 in normal rat islets diminished the change in mitochondrial membrane potential in response to glucose, reduced cytoplasmic ATP content, GSIS and mitochondrial ROS production [65,66]. *β*-cells exposed to free fatty acids displayed a lower mitochondrial membrane potential (less electronegative) and a decreased glucose-induced hyperpolarization. These effects were due to increased activity of UCP2 [67]. Conversely, UCP2-deficient mice demonstrated increased ATP production and improved GSIS [66]. In pancreatic islets from wild type but not *Ucp2*-knockout mice, genipin, a cell-permeant compound that was reported to inhibit UCP2-mediated proton leak, increased the mitochondrial membrane potential and cytosolic ATP, closed K_ATP _channels, and stimulated insulin secretion [68].

Our simulation gave similar results (Figure 4), showing that at a constant glucose level, increased uncoupling protein activity leads to decreases in Ψ_m_, the ATP/ADP ratio and [Ca^2+^]_c_. However, our simulation also shows a novel aspect of this problem: an ability of uncoupling proteins (and other uncoupling agents) to shift the glucose dependence of the ATP/ADP ratio and [Ca^2+^]_c_. This shifting mechanism is not simply varying the rates of a few processes. In our model, the shifting mechanism is generally characterized by small changes in Ψ_m_, ATP/ADP ratio or [Ca^2+^]_c _at low and high glucose levels. However, larger changes of [Ca^2+^]_c _can be expected in the region of physiological glucose concentrations (Figure 4), a hypothesis that needs to be further tested.

The shifting set-point mechanism would regulate insulin secretion particularly during a fluctuating nutrient supply. For example, UCP2 has been shown to be both induced and activated by exposure of rodent islets to high free fatty acids that cause mild uncoupling [64,67,69,70]. According to our analysis uncoupling can lead to a right shift in the glucose dependence of the ATP/ADP ratio and [Ca^2+^]_c_. This shift can result in decreased insulin secretion at moderate levels of glucose and at high levels of free fatty acids in blood. It may be a mechanism for restricting glucose consumption under conditions of increased serum fatty acid concentrations, when muscle cells can use free fatty acids as fuel rather than glucose.

Interestingly, this may be a physiologically important mechanism to blunt insulin production in starved animals even without increasing free fatty acid concentration. During starvation homeostatic mechanisms attempt to maintain a minimal acceptable blood glucose concentration, in part to maintain neurons that cannot use free fatty acids. Starvation induces UCP2 expression and reduces cellular NADH generation in response to glucose in mouse *β*-cells that would limit insulin secretion to reduce glucose uptake in muscle and adipose cells [71].

Our analysis supports an important role for uncoupling agents in *β*-cells that can coordinate the appropriate response of *β*-cells to fluctuating nutrient supply (see for example [24,63]. However, a pathological side effect might also accompany shifting ATP/ADP ratio and [Ca^2+^]_c _sensitivity to glucose, because decreased sensitivity of insulin secretion in response to glucose is a characteristic property of Type 2 diabetes [27,37]. Simulation with this model supports the idea that either an under or over-expression of UCP2 may lead to a failure of *β*-cells to properly respond to glucose.

### Role of Lactate dehydrogenase (LDH) and lactate production

Since *β*-cells have low levels of LDH (see Introduction) [1,14,15], we included a low level of LDH activity under basal conditions (Appendix). This led to a small lactate flux within the cytoplasm at low and medium glucose levels in our simulations (Figure 2A). However, the simulated lactate production increased significantly in response to high glucose. This can be accounted for by increased [NADH]_c_/[NAD^+^]_c _(see Equation 3, Appendix).

**Figure 5:**
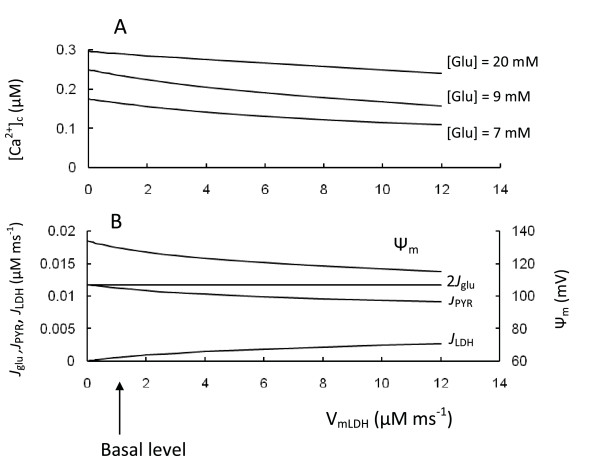
**Model parameters in response to changes of lactate dehydrogenase activity (V_LDH_)**. A. [Ca^2+^]_c _at different glucose levels. Low glucose level was 7 mM, medium was 9 mM and high was 20 mM; B. Dependence of Ψ_m_, *J*_pyr _and J_LDH _at a medium glucose concentration ([Glu] = 9 mM). Arrow corresponds to V_LDH _(Equation 3) for basal level of coefficient (Table 3). All other parameters were set as in Figure 2.

We also simulated a rise in LDH activity (Figure 5). This led to accelerated conversion of pyruvate to lactate, that decreased NADH production in mitochondria from pyruvate and the corresponding [NADH]_m_, Ψ_m_, ATP/ADP ratio as well as [Ca^2+^]_c _in response to increased glucose. This was all a consequence of the increased fraction of the glycolytic flux that was directed to lactate production.

Our simulations confirm that low levels of LDH expression in insulin-secreting cells are important for the correct channeling of pyruvate towards mitochondrial metabolism (see [1,16]). However, we also found that net lactate production increases significantly when extracellular glucose is increased (see Figures 2 and 5). For this reason, even low LDH activity can be an effective safeguard to prevent mitochondrial overexcitation at high glucose levels, where [Ca^2+^]_c _concentration is already saturated and increased Ψ_m _can lead to increased ROS production (see below).

Interestingly, overexpression of LDH in single MIN6 *β*-cells diminished their response to glucose as measured by mitochondrial NAD(P)H, Ψ_m_, cytosolic free ATP and Ca^2+ ^and led to a right shift in the glucose response of insulin secretion [16], all of which are simulated by our model. Since islet levels of LDH were increased in a rat pancreatectomy model of type 2 diabetes [72] and *β*-cell lines such as INS-1 have increased LDH activity that can partially explain their decreased sensitivity to glucose [14], overexpression of LDH may also be a possible mechanism of *β*-cell failure in specific cases.

### Role of NADH in cytoplasm and shuttle activity

The free cytoplasmic [NAD^+^]_c_/[NADH]_c _ratio can vary from about 700:1 to 200:1 in rat liver [73]. Zhang et al. [74] measured free [NAD^+^]_c_/[NADH]_c _in Cos7 cells and found a ratio of 644. Patterson et al. [47] found that the NAD(P)H concentration (NADH plus NADPH) was much lower in the cytoplasm than in mitochondria in pancreatic islets. Our calculations for the basal [NAD^+^]_c_/[NADH]_c _ratio level (Figure 2B) yield a magnitude of about 750:1 (at a glucose of 9 mM). Generally, our simulated [NAD^+^]_c_/[NADH]_c _ratios were in the range of experimentally observed values. As free NAD^+ ^levels greatly exceed those of NADH, large changes in the [NAD^+^]_c_/[NADH]_c _ratio do not require correspondingly large changes in free [NAD^+^]_c_. Thus, changes in cytoplasmic redox level could be manifested primarily through variation in [NADH]_c_.

**Figure 6:**
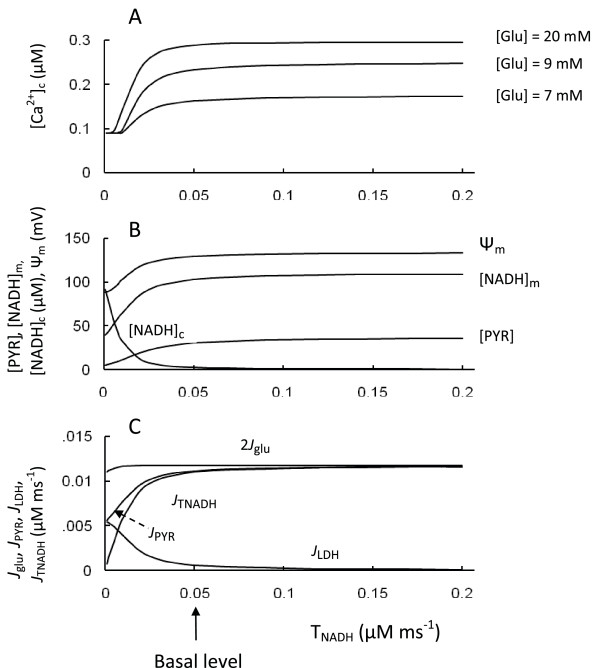
**Model parameters in response to changes of the transport rate coefficient for NADH shuttles (T_NADH_)**. A: [Ca^2+^]_c _at different glucose levels. B: Ψ_m_, [PYR], [NADH]_c _and [NADH]_m _at medium glucose level (9 mM); C: 2 *J*_glu_, *J*_PYR_, *J*_LDH _and *J*_TNADH _at medium glucose level (9 mM). Arrow corresponds to T_NADH _(Equation 9) for basal level of coefficients (Table 3).

Pyridine nucleotides are not only key molecules for metabolic conversions, but also can serve as critical signaling molecules, such as the cytoplasmic NAD(P)H/NAD(P)^+ ^ratio [2,42,53,75]. Here we have focused only the energetic aspects of pyridine nucleotides and modeling. Generally, redox shuttles are responsible for maintaining and restoring cytoplasmic NAD(P)H/NAD(P) ^+ ^ratios and the cytoplasmic/mitochondrial redox state in pancreatic *β*-cells (see Introduction). NADH generated by glycolysis was efficiently reoxidized by highly active mitochondrial shuttles rather than by lactate dehydrogenase in basal conditions in our model (flux *J*_TNAD _is considerably higher than *J*_LDH _in Figure 2A). Steady-state simulation was designed to investigate a role of the redox shuttles in cytoplasmic and mitochondrial events following a step change in the transport rate coefficient for NADH shuttles at several glucose levels (Figure 6).

The result of this simulation showed that [Ca^2+^]_c _quickly reached saturation with increased shuttle activity from the basal level. However, a decrease in transport rate coefficient (T_NADH_) resulted in a decrease in ATP/ADP ratio and in [Ca^2+^]_c _from the apparent threshold (Figure 6A). Failure to activate NADH-NAD^+ ^transport between the cytosol and mitochondria does not significantly alter the mitochondrial [NAD^+^]_m_/[NADH]_m _ratio but significantly increases the cytosolic [NADH]_c_/[NAD^+^]_c _ratio, since [NADH]_c _increases quickly with decreased T_NADH _(Figure 6B). Increasing the [NADH]_c_/[NAD^+^]_c _ratio led to an acceleration of lactate production from pyruvate (Equation 9, Appendix). The corresponding reduction in pyruvate concentration decreased mitochondrial ATP production, leading to a decreased ATP/ADP ratio and decreased [Ca^2+^]_c_. However, glucose consumption did not decrease significantly, because excess glucose influx was shifted to accelerated lactate production in the cytoplasm, as a consequence of the sharp increase in the cytoplasmic NADH concentration (Figure 6). This *in silico *study shows that the cytosolic NADH transport into the mitochondria can be a key regulator of cytosolic NADH/NAD^+ ^and lactate production in pancreatic *β*-cells only when its influx rate was considerably decreased from basal conditions. Similar results were obtained in a simulation of the shuttle system in cardiomyocytes [76].

The model simulations on the role of shuttles are also similar to published data. For example, in islets deficient in glycerol-3-phosphate dehydrogenase (GPDH) (which would effectively eliminate the glycerol-phosphate shuttle), when the malate-aspartate shuttle was blocked by inhibiting aspartate aminotransferase by aminooxyacetate, glucose-induced increases in cellular ATP content were impaired and insulin secretion was eliminated, whereas the glycolytic flux remained unchanged [19]. However, studies of this mGPDH-/- mouse model also show that neither absence of the glycerol-phosphate shuttle (in mGPDH-/- islets) nor suppression of the malate-aspartate shuttle alone (in wild-type islets) altered ATP synthesis or GSIS [19]. Our simulations (Figure 6) suggest an explanation for these interesting results. Decreased shuttle activity did not lead to a change in [Ca^2+^]_c_, if the T_NADH _initial value was close to a saturated level, and only significant inhibition of shuttle activity, when both shuttles are blocked, resulted in a decrease of [Ca^2+^]_c_, whereas the glucose consumption remained unchanged.

The effects of agents known to increase shuttle activities were also examined. Shuttle agonists increased the average [Ca^2+^]_c _in mouse islets in the presence of 12 mM glucose [18]. Adenoviral overexpression of the protein Aralarl, a Ca^2+ ^sensitive member of the malate-aspartate shuttle, in both insulin-secreting INS-IE cells and rat pancreatic islets, enhanced glucose-evoked NAD(P)H autofluorescence, Ψ_m _and insulin secretion. Glucose oxidation was enhanced and lactate production was reduced [77]. These experimental results are in reasonably good agreement with the simulated [Ca^2+^]_c_, Ψ_m _and [NADH]_m _increases and decreased lactate output (*J*_LDH_) in Figure 6 when T_NADH _was increased from the basal level.

Interestingly, activity and expression of the key enzymes of NADH shuttles were found to be significantly decreased in fetal rat and pig islets compared with adult islets. This can contribute to the inability of fetal *β*-cells to secrete insulin robustly in response to glucose [78]. Activity of mGPDH, the key enzyme in the glycerol phosphate shuttle, was reduced in islets from patients with type 2 diabetes [79], and it follows that decreased levels of NADH shuttle activity could also be a possible contributor to *β*-cell secretory failure [27]. Our simulations confirm the possibility that substantial decreases in activity of NADH shuttles can result in secretory failure of *β*-cells. In this case our model also predicts an increased lactate production rate in *β*-cells.

### Role of mitochondrial Ca^2+ ^handling in *β*-cells

Calcium signaling is associated with mitochondrial uptake of Ca^2+ ^[10]. Recent islet studies have shown steady state resting mitochondrial [Ca^2+^]_m _levels are relatively low. Calibrated resting [Ca^2+^]_m _in rat islets was approximately 250 nM at 3 mM external glucose. Increasing glucose to 16 mM resulted in a rise of cytoplasmic and mitochondrial [Ca^2+^] above its resting level [48,50]. The simulated increase of [Ca^2+^]_m _versus glucose concentration for steady-state (Figure 2E) was in agreement with these experimental data.

**Figure 7:**
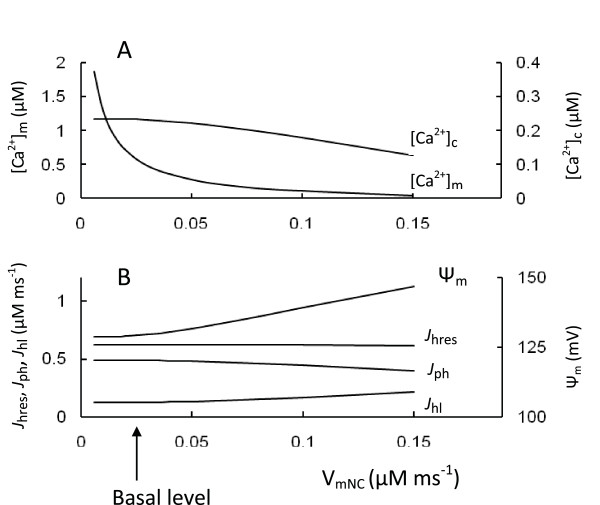
**Model parameters in response to changes of the maximal Na^+^/Ca^2+ ^antiporter rate (V_mNc_)**. A: [Ca^2+^]_c _and [Ca^2+^]_m_; B: respiration rate (*J*_hres_), phosphorylation rate (*J*_ph_), leak (*J*_hl_) and Ψ_m_. Arrow corresponds to V_mNc _(Equation 11) for basal level of coefficients (Table 3). Calculations were performed at medium glucose level (9 mM).

We explored the parameter space with respect to the mitochondrial Na^+^/Ca^2+ ^antiporter rate to simulate a change of [Ca^2+^]_m_. We simulated inhibition and activation of the Na^+^/Ca^2+ ^exchanger velocity at medium glucose level that resulted in changes in [Ca^2+^]_m _at steady-state (Figure 7). We analyzed several aspects of the possible role of [Ca^2+^]_c _in regulating mechanisms comparing of the experimental evidences and our simulations.

#### 1. Mitochondrial Ca^2+ ^as an accelerator of ATP production

Mitochondrial Ca^2+^controls the key rate-limiting steps in the TCA cycle through activation of pyruvate dehydrogenase and at least two TCA cycle enzymes: isocitrate dehydrogenase and *α*-ketoglutarate dehydrogenase (reviewed in [80]) and F_1_F_0 _ATPase [11]. A control hypothesis emerged from this discovery [81]. According to this hypothesis an increase in glucose concentration is accompanied by a rise in cytoplasmic Ca^2+^, and the subsequent effect of matrix Ca^2+ ^on the TCA cycle increases the supply of reducing equivalents (NADH, FADH2) leading to a "push" of electrons through the respiratory chain. This accelerates ATP production by generating more proton motive force, leading to increased [NADH]_m _and stimulating oxidative phosphorylation [9,81].

However, a dominant role of [Ca^2+^]_m _in a control of *β*-cell oxidative metabolism under physiological conditions is questionable. The initial mitochondrial NADH and Ψ_m _response precedes increased cytoplasmic and mitochondrial Ca^2+ ^in response to a sharp increase in glucose [3,20,21,50]. In addition, respiration rate and insulin secretion can initially follow the glycolytic rate, which is determined by glucokinase activity, rather than by [Ca^2+^]_m _increase (see [10]). For this reason, it was proposed that Ca^2+ ^is more involved in the maintenance rather than in the initiation of glucose metabolism-secretion coupling. At high glucose, glycolysis may become sufficiently fast such that pyruvate oxidation becomes rate limiting in the formation of ATP or glucose-derived intermediates [41,52]. Under this limitation, positive regulation of mitochondrial metabolism can require additional Ca^2+ ^activation for the synthesis of ATP and other coupling factors (see [10]).

Because [Ca^2+^]_m _can be regulated by mitochondrial Na^+^/Ca^2+ ^exchanger activity, inhibition of the Na^+^/Ca^2+ ^exchanger was suggested as a possible target to increase [Ca^2+^]_m _and thereby improve insulin secretion in type 2 diabetes. An inhibitor of the exchanger (CGP37157) was shown to prolong mitochondrial Ca^2+ ^signals and increase insulin secretion [82]. (However, this inhibitor could block plasma membrane Ca^2+ ^channels at high concentrations [83]).

Our model allows an evaluation of the influence of [Ca^2+^]_m _changes on GSIS. The results of simulations (Figure 7) showed that increasing mitochondrial [Ca^2+^]_m _by inhibiting the Na^+^/Ca^2+^antiporter did not initially lead to any changes in mitochondrial fluxes or the corresponding increase in the ATP/ADP ratio and [Ca^2+^]_c _at all glucose levels evaluated. The initial decrease of [Ca^2+^]_m _due to an increased maximal velocity of Na^+^/Ca^2+ ^also did not lead to significant changes in the ATP/ADP ratio and [Ca^2+^]_c _(Figure 7). We found that the reason for such insensitivity to [Ca^2+^]_m _was that [Ca^2+^]_m _was above the threshold for activation of mitochondrial processes even at basal conditions. Our simulations showed that the respiration rate and insulin secretion may follow the glycolytic rate at physiological conditions, rather than an increase in [Ca^2+^]_m _(see above).

However, a large decrease in [Ca^2+^]_m _due to a large increase in the maximal velocity of Na^+^/Ca^2+ ^exchange led to an inhibition of ATP production and a decreased ATP/ADP ratio and [Ca^2+^]_c _(Figure 7). Our simulations also suggest that an increase in GSIS can be expected following increased [Ca^2+^]_m_, for example, following inhibition of the Na^+^/Ca^2+ ^exchanger but only if the initial [Ca^2+^]_m _is so low as to limit mitochondrial reactions. The effect of [Ca^2+^]_m _on activation of mitochondrial processes in *β*-cells should therefore be further tested specifically under hyperglycemic and hyperlipidemic conditions.

#### 2. Mitochondrial Ca^2+ ^influx as a suppressor of ATP production

Physiological influx of Ca^2+ ^into the mitochondrion can cause a measurable concurrent mitochondrial depolarization [84]. Ca^2+ ^cycling in mitochondria reflects the uptake of Ca^2+ ^electrogenically coupled to the efflux of Ca^2+ ^in exchange for protons or sodium ions. This effectively results in uncoupling and would be included in proton leak measurements (Equation 18, Appendix). A high mitochondrial Ca^2+ ^influx and efflux equivalent to an increased proton leak would cause a fall in membrane potential. This would shut down ATP production by the F_1_F_0 _ATPase, a process referred to as "short circuiting" in the several mathematical models for *β*-cell mitochondria in [29,30,85]. In these models the uptake of Ca^2+ ^by *β*-cell mitochondria suppressed the rate of production of ATP via oxidative phosphorylation.

However, the conclusion that mitochondrial Ca^2+ ^cycling leads to energy dissipation in the pancreatic *β*-cell is not supported by experimental data which actually favors the opposite idea, that the primary role of mitochondrial Ca^2+ ^is the stimulation of oxidative phosphorylation [9]. The contribution of Ca^2+ ^cycling to proton leak was estimated to be only about 1% of the state 3 rate [9,86]. Mitochondrial Ca^2+ ^cycling also does not lead to marked energy dissipation in the heart [87]. Recordings in mouse islet cells revealed no effect of the inhibitor of the Na^+^/Ca^2+ ^exchanger (CGP-37157) on the hyperpolarized Ψ_m _under glucose-stimulated conditions [83] suggesting that mitochondrial Ca^2+ ^cycling likely does not make a major contribution to energy dissipation. Our simulation shows increased ATP production coincidentally with increased [Ca^2+^]_c _does not decrease Ψ_m _(Figure 2). The contribution of Ca^2+ ^cycling to the fluxes that result in a dissipation of Ψ_m _was less than 1% in our model. This is due to low activity of the Ca^2+ ^uniporter and Na^+^/Ca^2+ ^exchanger that we have employed to approximate the observed delay between the oscillations [Ca^2+^]_c _and [Ca^2+^]_m _in *β*-cells *in vivo *(see Appendix). The overestimation of the role of Ca^2+ ^fluxes in the dissipation of Ψ_m _in *β*-cell models [28-30,85] may have arisen from the overestimation of mitochondrial Ca^2+ ^fluxes taken from data obtained on isolated mitochondria.

#### 3. Regulation of cytoplasmic Ca^2+ ^concentration by [Ca^2+^]_m_

The role of mitochondrial Ca^2+ ^handling in the regulation of cytoplasmic Ca^2+ ^concentration has been emphasized in several cell types [9]. Mitochondria are an important storage component for Ca^2+ ^handling in cardiomyocytes, where fast and large cytoplasmic Ca^2+ ^changes define cardiac excitation-contraction coupling [87,88]. However, an estimation of the subcellular compartmental volumes of cardiomyocytes gave 58.5% cytosolic and 36% mitochondrial volumes, respectively [89]. On the other hand, *β*-cell mitochondrial volumes ranged from only 4% to 8% per cell [90,91]. This greatly limits the degree to which mitochondria can regulate cytoplasmic Ca^2+ ^concentration in *β*-cells.

Ca^2+ ^influx through L-type Ca^2+ ^channels and efflux through plasma membrane pumps, along with endoplasmic reticulum (ER) stores are the principal regulators of *β*-cell cytoplasmic Ca^2+ ^homeostasis [4,5,10,92,93]. A robust mitochondrial Ca^2+ ^pool was not a necessary component of our previous model examining regulation of cytoplasmic Ca^2+ ^homeostasis (see [5,26]). The mitochondrial Ca^2+ ^pool is significantly smaller compared with the ER free Ca^2+ ^pool, because the ER volume can be up to 20% of total *β*-cell volume [90] and its free Ca^2+ ^concentration can reach several hundred mM (see Refs. [4,5,92]). Given the smaller mitochondrial volume, our simulations did not show significant Ca^2+ ^fluxes between cytoplasm and mitochondria (see above). This raises the question as to whether *β*-cell mitochondrial Ca^2+ ^handling can play a significant role in the regulation of *β*-cell [Ca^2+^]_c _under physiological conditions.

### Variations in mitochondria operation rates and content

Reduction in mitochondrial metabolism and/or cellular content (number or volume) can in principle underlie progression to the decreased insulin secretion typical of Type 2 diabetes [35,37,94]. For this reason, we employed our model to simulate the effect of changes in mitochondrial functional activity and/or content.

#### 1. Suppression of respiratory activity

**Figure 8:**
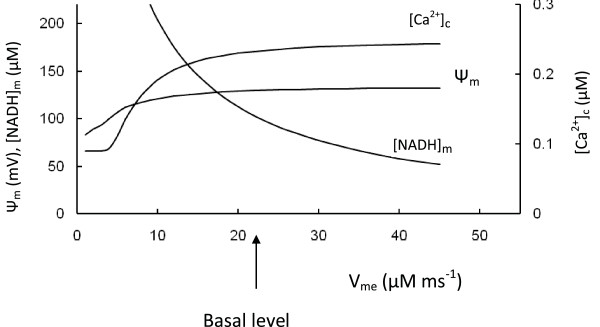
**Model parameters in response to changes of the maximal rate of proton pumping in ETC (V_me_)**. [Ca^2+^]_c_, Ψ_m _and mitochondrial NADH concentration were calculated for medium glucose level (9 mM). Arrow corresponds to the basal V_me _(Table 3).

We varied the maximal rate of ETC proton pumping (V_me_) (Equation 5, Appendix) to simulate of the results of experiments where the electron transport chain activity was decreased (Figure 8). As expected, this simulation showed an increased [NADH]_m _with decreased V_me_, however, Ψ_m_, ATP production, respiration rate, ATP/ADP ratio and [Ca^2+^]_c _were also decreased. The glucokinase reaction rate was not changed because the rate of pyruvate reduction by LDH was increased as a consequence of increased [Pyr]. This was due to increased [Pyr] and the part of glycolytic substrate that was not used for ATP synthesis was removed by increased lactate production (not shown).

These simulations correspond to data [95-97] obtained following inhibition of transcription of mitochondrial DNA by ethidium bromide (EtBr), leading to a reduced expression of the mitochondrial electron transport system. After EtBr treatment (in the INS-1 *β*-cell line), Ψ_m_failed to hyperpolarize in response to glucose and ATP production and insulin secretion were significantly decreased [97]. Noda et al. [96] found EtBr caused increased NADH accumulation and lactate production in *β *HC9 cells, along with decreased [Ca^2+^]_c _and ATP/ADP ratio. In contrast, glucose utilization was only insignificantly decreased.

#### 2. Suppression of F_1_F_0 _ATPase activity

**Figure 9:**
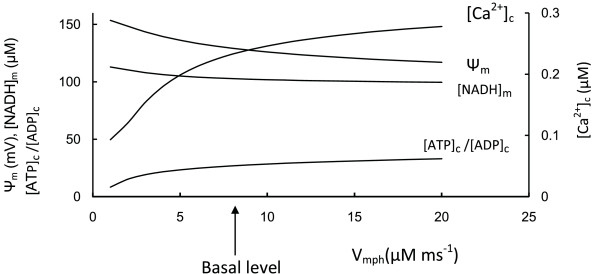
**Model parameters in response to changes of the maximal rate of proton flux in F_1_F_0 _ATPase (V_mph_)**. [Ca^2+^]_c_, Ψ_m_, [ATP]_c_/[ADP]_c _and mitochondrial NADH concentration were calculated for medium glucose level (9 mM). Arrow corresponds to the basal V_mpe _(Table 3).

We varied also the maximal F_1_F_0 _ATPase activity (V_mph_) (Appendix, Equation 7) to simulate of the results of experiments where this activity was changed. Suppression of F_1_F_0 _ATPase activity resulted in decreased of ATP synthesis in mitochondria. This decreased [ATP]_c _and increased [ADP]_c _leading to a decreased ATP/ADP ratio and decreased [Ca^2+^]_c _(Figure 9). Decreased proton flux rates through the F_1_F_0 _ATPase led to an increase in Ψ_m_. According to Equation 5C an increase in Ψ_m _decreases ETC activity. This decreased [NADH]_m _consumption lead to decreased respiration rate (measured as O_2 _consumption) (simulations not shown) and insignificant [NADH]_m _accumulation. Interestingly, here as well as in first case, the rate of glucokinase reaction was not changed because the NADH not utilized for ATP production was expended on increased leak due to increased Ψ_m _(not shown).

These simulations correspond to the data obtained on BHE/cdb rats, which have a mutation in ATP synthase that limits ATP production and leads to development of mild diabetes [98,99]. BHE/cdb rat islets showed reduced responsiveness to glucose stimulation and ATP content was lower than in control islets [99]. The authors suggested that Ψ_m _is increased in BHE/cdb rat islets due to increased oxygen radical formation [99]. GSIS was reduced, but could not be attributed to changes in glucokinase activity or islet glucose uptake [100].

#### 3. Changes in mitochondria activity or content

**Figure 10:**
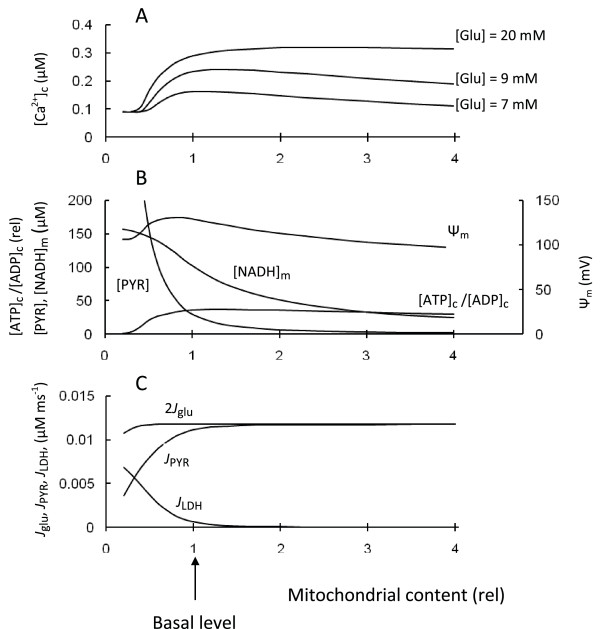
**Steady-state simulations of parameters in response to changes of mitochondria content**. We simultaneously changed all maximal rates of mitochondrial processes (*J*_PYR_, *J*_hres_, *J*_ph_, *J*_hl_, j_TNADH_, *J*_uni_, *J*_NCa_)- The arrow corresponds to the basal maximal rates and mitochondrial volume from Tables 2 and 3 (shown on axis as 1). A: [Ca^2+^]_c _at different glucose levels. B: Ψ_m_, [PYR], [NADH]_c _and [ATP]_c_/[ADP]_c _at medium glucose level (9 mM); C: 2 *J*_glu_, j_PYR _and J_LDH _at medium glucose level (9 mM). All other parameters were set as in Tables 2 and 3.

To simulate changes in mitochondrial activity or content we varied the mitochondrial volume and corresponding maximum rate of the mitochondrial reaction fluxes in the model (Figure 10). The simulation showed that decreased maximal rates of all intramitochondrial processes from the basal level, corresponding to decreased mitochondrial activity or content per cell volume, resulted in increased pyruvate and [NADH]_m _concentrations, coincidentally with a decreased Ψ_m_, ATP/ADP ratio and [Ca^2+^]_c _(Figure 10A,B). The glucose consumption rate decreased moderately since the increase in [PYR] led to an increase in lactate production (Figure 10C) that together with a decreased rate of pyruvate decarboxylation in mitochondria led to an insignificant change in glycolytic flux.

These model simulations for decreased mitochondrial activity or content are in accordance with experimental data. For example, a pancreatic *β*-cell mouse model for mitochondrial diabetes induced by tissue-specific disruption of the nuclear gene encoding the mitochondrial transcription factor A (Tfam) displayed severe mtDNA depletion, deficient oxidative phosphorylation and abnormally enlarged islet mitochondria [101]. Tfam is essential for mtDNA expression and maintenance, *β*-cell stimulus-secretion coupling in isolated islets from tfam -/- mice showed reduced hyperpolarization of the mitochondrial membrane potential, impaired Ca^2+^-signaling and lowered GSIS [101]. Similarly, *β*-cell specific disruption of Tfam led to 50% reduction in mRNA levels for the mitochondrially encoded *nd1 *gene, a subunit of the NADH dehydrogenase comprising complex I of the mitochondrial respiratory chain. As a consequence, total cellular ATP concentration was drastically decreased by 75%, and glucose failed to augment cytosolic ATP, explaining the blunted GSIS [102].

Simulation of increased mitochondrial content above basal levels leads to an initial increase in [Ca^2+^]_c _at the tested glucose levels. This initial increase of [Ca^2+^]_c _is due to increased ATP production (and an increase in ATP/ADP ratio) as a result of decreased lactate production (see Figure 10C). Interestingly, our simulation shows decreased [Ca^2+^]_c _following increased mitochondrial content, due to decreased ATP production (with decreased ATP/ADP ratio) at low and medium glucose levels. This is a result of increased total leak activity that occurs in the model as a consequence of increased mitochondrial content. According to the simulation, lactate production was higher and relative leak was lower (compared with electron transport rate) in basal conditions at high glucose content. For this reason [Ca^2+^]_c _did not decrease with increased mitochondrial activity or content at high glucose level ([Glu] = 20 mM) (Figure 10A).

While our simulations support the obvious result that a decrease in mitochondrial function (or content) leads to decreased ATP production, ATP/ADP ratio and [Ca^2+^]_c _response to glucose, together giving decreased glucose sensitivity (Figure 8, 9, 10), the simulations also show that subtle variation in mitochondrial function or content could underlie *β*-cell defects in type 2 diabetes (see [10,37,103]. On the other hand, increased mitochondrial content can, in theory, initially increase *β*-cell sensitivity to glucose (Figure 10) especially if their initial content was decreased in comparison with basal levels. This supports the idea that an increase in mitochondrial content can be a possible target for treatment of type 2 diabetes.

#### Oscillation processes

Oscillations in cytoplasmic and mitochondrial metabolism, membrane potential, intracellular and mitochondrial Ca^2+ ^due to increased glucose concentrations has been described as a specific characteristic of glucose signaling in the *β*-cell [3,20-22,104]. The source of these oscillations and the mechanism of orchestration are not clearly understood and may reflect multiple processes [5,23,26,105]. Particularly, [Ca^2+^]_c _oscillations can be the driving force for other oscillations in pancreatic *β*-cells (see [26,106]). For this reason, the aim of this section is testing the hypothesis that independent [Ca^2+^]_c _oscillations can create coupled oscillations in metabolic processes.

**Figure 11:**
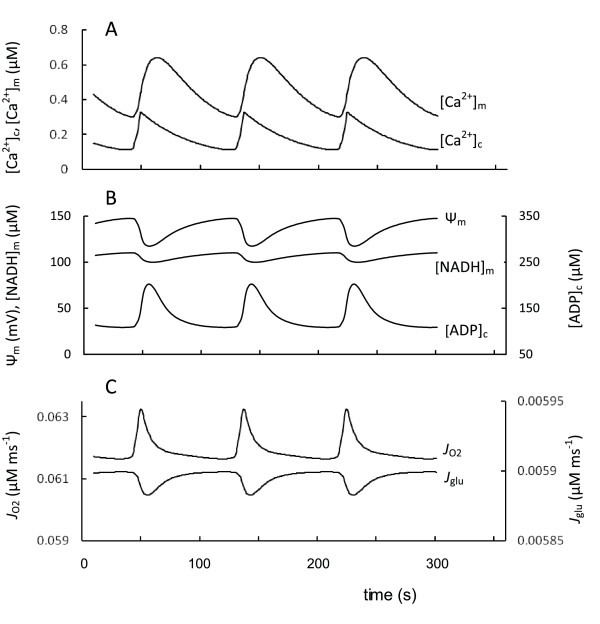
**Model-predicted dynamic responses of parameters in pancreatic *β*-cells for independent [Ca^2+^]_c _oscillations at 9 mM glucose concentration**. A: independent [Ca^2+^]_c _oscillations (Appendix) and simulated [Ca^2+^]_m _transient in response to [Ca^2+^]_c _oscillations; B: Ψ_m_, [ADP]_c _and [NADH]_m_; C: glucose consumption (*J*_glu_, Equation 1) and oxygen consumption (*J*_O2_, Equation 6). All other parameters set points were taken for basal conditions (Tables 2 and 3). Variations in the oxidative phosphorylation rate (as *J*_ph_) were determined in our model mainly by the [ADP]_c _changes (Equation 7) and this rate increased with [ADP]_c _increase following [Ca^2+^]_c _rise, that in turn decreased Ψ_m _and increased O_2 _consumption rate.

We previously developed a mathematical model describing *β*-cell ion regulation that shows how [Ca^2+^]_c _oscillations can be independently established from mitochondrial processes when the ATP/ADP ratio achieved some threshold leading to initial depolarization of the plasma membrane [5]. However, for ease of use here we employed a simplified mathematical model that created a periodically varied [Ca^2+^]_c _in the cytoplasm (Equations 24-30, Appendix) (Figure 11A). Using this simpler model we simulated the characteristic shape of slow [Ca^2+^]_c _oscillations (with a period of one minute and longer), where [Ca^2+^]_c _increased sharply and the quiescent time was longer than the period with increased [Ca^2+^]_c _(the experimental examples of such oscillations in *β*-cells are shown [5,23,106]). Our model simulated the corresponding changes in several *β*-cell mitochondrial and metabolic processes (Figure 11). This simulation showed that the metabolic and membrane variables in the cytoplasm and mitochondrial matrix can display oscillatory behavior when [Ca^2+^]_c _oscillated independently.

A mechanism involving periodic nucleotide concentrations links [Ca^2+^]_c _changes to activation of metabolic oscillations. Each [Ca^2+^]_c _increase during oscillations leads to increased cytoplasmic ATP consumption (Equation 20, Appendix). This decreases [ATP]_c _and increases [ADP]_c _(Figure 11B) leading to a decreased ATP/ADP ratio. This resulted in an amplification of ATP synthesis by the mitochondrial F_1_F_0 _ATPase (Equation 7A). Increasing the rate of proton flux through F_1_F_0 _ATPase led to a decrease in Ψ_m _(Figure 11B). According to Equation 5C (Appendix) a decrease in Ψ_m _increases ETC activity. This enhanced [NADH]_m _consumption and the respiration rate, measured as O_2 _consumption. For this reason, the electron transport and respiration rates were substantially in phase with [Ca^2+^]_c _oscillations (Figure 11C). The oscillations in the glucose consumption rate determined by glucokinase (Equation 1, Appendix), that follows the [ATP]_c _changes, were out of phase with [Ca^2+^]_c _oscillations (Figure 11C).

The experimental data are in accordance with our model simulations. For example, increased [Ca^2+^]_c _in MIN6 cells at constant glucose levels caused a fall in the ATP/ADP ratio as inferred from an experiment tracking luciferase-generated photons in transfected cells expressing luciferase [13]. Glucose-induced NAD(P)H and [Ca^2+^]_c _slow oscillations were measured simultaneously in mouse pancreatic islets, revealing that NAD(P)H oscillations were small and preceded those of calcium by about 0.1 of a period [21]. In our model the mitochondrial NADH peak concentrations also slightly preceded [Ca^2+^]_c _periodic maxima (see Figures 11A,B). The delay (about 9 sec) was about 0.1 of a period of simulated [Ca^2+^]_c _oscillations.

Glucose-induced [Ca^2+^]_c _and Ψ_m _slow oscillations have been reported [20-22,104], and measured simultaneously in mouse pancreatic islets [20]. The results (Figure 4 from Ref. [20]) were similar to the dependence that we calculated (Figures 11A,B).

Oscillations in islet oxygen and glucose consumption have also been recorded [95,107]. The glucose consumption rate was out of phase with slow [Ca^2+^] oscillations, and oxygen consumption rate and [Ca^2+^]_c _changes were approximately in phase. The mechanism of this phenomenon is as yet unknown. However, these data are in accord with our model simulation (Figure 11A,C) and could be explained by decreased ATP and increased free ADP concentrations with increased [Ca^2+^]_c _during the appropriate phase of slow oscillations.

Mechanisms other than [ADP]_c _changes can cause variations in [Ca^2+^]_m_. Because the changes of Ψ_m _were modest while [Ca^2+^]_c _varied with significant amplitude during oscillations, an influx of Ca^2+ ^into mitochondria is determined mainly by a change in [Ca^2+^]_c _while Ca^2+ ^efflux depended predominantly on [Ca^2+^]_m _changes in our model. This led to some delay in [Ca^2+^]_m _oscillations in comparison with [Ca^2+^]_c _(14 sec in Figure 11A). The value of this delay was used here to determine maximal rates of Ca^2+ ^fluxes (P_Ca _in Equation 10 and V_NC _in Equation 11, Appendix).

These results of simulations were consistent with observations demonstrating that oscillations in mitochondrial Ca^2+ ^were in response to glucose elevations, presumably tracking oscillations in [Ca^2+^]_c _[50,82,108,109]. Periodic oscillations in [Ca^2+^]_m _followed [Ca^2+^]_c _oscillations with a delay of approximately 14 sec [50,109]. A similar delay between the maxima of [Ca^2+^]_c _and [Ca^2+^]_m _(14 sec) was simulated in Figure 11A.

From these results, our simulations at least partially confirm a possible cycle of events previously suggested whereby increased [Ca^2+^]_c _during oscillations results in a decrease in ATP/ADP ratio due to increased ATP consumption [13,50]. In the next phase, when [Ca^2+^]_c_decreases, ATP production outweighs ATP consumption leading to an increasing ATP/ADP ratio. However, this pathway is unlikely to be a possible pacemaker mechanism for [Ca^2+^]_c _oscillations as has been proposed [13,50]). Rather this could serve as a mechanism where cyclic changes in ATP/ADP ratio are determined by independent cytoplasmic Ca^2+ ^oscillations.

Oscillations in Ψ_m _and mitochondrial NADH are usually small in amplitude in pancreatic *β*-cells [20-22,104]. Mitochondrial Ca^2+ ^oscillations, on the other hand, are reasonably large.

However, they have a typical dynamic that is intrinsic to two successive components where [Ca^2+^]_m _follows [Ca^2+^]_c _with a particular delay (see above). Our dynamic simulations also clearly show that the independent [Ca^2+^]_c _oscillations lead to simulation of [Ca^2+^]_m_, Ψ_m_, mitochondrial NADH and respiration oscillations that were similar to experimental observations. These experimental data and our simulations suggest that independent [Ca^2+^]_c _oscillations can be a pacemaker in the generation of oscillations of mitochondrial and cytoplasmic parameters in *β*-cells.

### Regulation of ROS content in *β*-cells

In most cells mitochondria represent the main source of the physiological production of reactive oxygen species (ROS), which may be a byproduct of ETC function [9,110], while recent evidence suggests that NADPH oxidase-dependent generation of ROS in pancreatic *β*-cells [111] and ROS generation from sulfhydryl formation in proinsulin biosynthesis [112] are also potentially important potential sources of ROS generation.

ROS production in mitochondria depends upon the redox state of the ETC complexes, since the ETC carriers in a reduced state have the property of donating electrons to oxygen [110]. The redox state of the ETC complexes and, consequently, the rate of superoxide production also highly depend on Ψ_m_. The increased Ψ_m _(above about 160 mV) decreases electron transport capability leading to a reduced state of the carriers and sharply increases ROS production [110,113]. A pronounced increase in Ψ_m _also augmented ROS production in pancreatic *β*-cells [36,70].

Interestingly, *β*-cells have relatively low levels of free radical detoxifying and redox-regulating enzymes such as superoxide dismutase, glutathione peroxidase, catalase and thioredoxin [114,115]. The reasons for this are unclear, although it has been suggested ROS may also subserve a signaling function [116]. However, despite the reported low expression of detoxifying and redox-regulating enzymes in *β*-cells, antioxidant systems appear to be sufficient to prevent acute oxidative damage under normal physiological conditions [114,115]. We can explain this intriguing property of *β*-cells based on our simulations results, which show (Figures 2 and 3) that *β*-cells usually work at a relatively lower Ψ_m_(< 160 mV) compared with other types of cells (see above). This leads to decreased ROS production and, consequently, *β*-cells require a decreased level of detoxifying and redox-regulating enzymes, at least under "normal" conditions, as has been reported.

However, a decreased level of detoxifying and redox-regulating enzymes may also contribute to increased sensitivity of *β*-cell mitochondria to damage when ROS production increases above normal levels. For example, as was pointed out above, increased respiratory activity, decreased UCP content, decreased LDH activity or high glucose levels could lead to increased Ψ_m _and subsequently to increased ROS production. The imbalance in ROS production versus protection can occur in *β*-cells more easily than in other types of cells because of decreased level of detoxifying and redox-regulating enzymes.

For these reasons, an overload by metabolic secretagogues such as glucose, causing increased insulin secretion following increase in Ψ_m _and ATP/ADP ratio can also induce increased oxidative stress as a result of elevated ROS production and decreased ability for defense. This could quickly result in ROS-induced reduction in mitochondrial function and cellular content, decreased ATP synthesis, dysregulated calcium homeostasis and ultimately cell death. Decreased mitochondrial functional activity or content could lead to decreased glucose sensitivity and consequently can lead to type 2 diabetes [36,37,117].

## Conclusion

The integrated mathematical model of *β*-cell energetics constructed here has allowed us to reproduce and suggest explanations for experimental relationships among Ψ_m_, respiration, NADH, mitochondrial and cytoplasmic Ca^2+ ^and ATP/ADP ratio and other parameters under various conditions. Our analysis shows that the control structure of *β*-cell energetics is determined predominantly by the need to regulate the ATP/ADP ratio to keep sensitivity to glucose within the physiological range.

Special features of glucose metabolism in pancreatic *β*-cells are central to enable this physiological role. Three of these characteristics were emphasized in our model: the glucose phosphorylation by the high-K_m _glucokinase, which is rate-limiting for glucose-sensitive metabolism and determines the glucose dependency curves of many processes in the *β*-cell; the remarkably low activity of LDH; and the presence of effective hydrogen shuttles to allow virtually quantitative oxidation of glycolytic NADH (see above). Our analysis is both influenced by and supports these proposals.

We found that the adaptive mechanisms in mitochondria can include decreased respiratory activity compared with F_1_F_0 _ATPase activity. This mechanism leads to mitochondrial function in the region with decreased Ψ_m _where variations in Ψ_m _should result in high sensitivity to physiological glucose levels and low ROS production. We found also that proton leak allows fine adjustment and exerts a higher level of control by shifting the ATP/ADP ratio and [Ca^2+^]_c _sensitivity to glucose.

This comprehensive model of *β*-cell energetics permits testing of bioenergetic control hypotheses, provides a basis for further refinement of such steps as oxidative phosphorylation, mitochondrial Ca^2+ ^handling and allow for future incorporation of other biochemical pathways.

## Appendix

### Model of energetic and mitochondrial processes

The reaction network of the model is shown in Figure 1. Due to the large number of reactions under consideration, the mathematical description is simplified as much as possible and in some cases intermediate metabolites were lumped together. To help simplify the mitochondrial currents and variables, we represented respiration and ATP synthesis rates in terms of effective proton fluxes.

The parameters for the model are given in Tables 2 and 3 along with references to the specific original studies. However, to achieve a closer correspondence with metabolism in intact *β*-cells or islets we have modified some of these parameters to more closely reflect the complex experimental measurements *in vivo*, where indicated.

**Table 2:** Standard physical and cellular parameter values (see text for explanations)

Parameters	Symbol	Value	Units	Reference
Relative cytoplasmic volume per *β*-cell	V_i_	0.53	Ul	[90]
Relative mitochondrial volume per *β*-cell	V_mit_	0.06	Ul	[90,91]
Relative mitochondrial matrix volume	V_mmit_	0.0144	Ul	Ad
Mitochondrial membrane capacitance for *β*-cells	C_mit_	1.812	μM mV^-1^	[136]
Faraday's constant	F	96484.6	C mole^-1^	Physical constant
Valence of Ca^2+^	ZCa	2	Ul	Physical constant
Fraction of free Ca ^+ ^in Mitochondria	f_i_	0.01	Ul	[5]
Fraction of free Ca^2+ ^in mitochondria	fm	0.0003	Ul	[28]
Cytoplasmic Na^+ ^concentration	[Na^+^]_c_	10000	*μ*M	[130]
Mitochondrial Na^+ ^concentration	[Na^+^]_m_	5000	*μ*M	[130]
Thermal voltage (RT/F) (37°C)	T_v_	26.73	mV	Physical constant
Cellular adenine nucleotides concentration	A_t_	4000	*μ*M	[5]
Free pyridine nucleotides concentration in mitochondrial matrix	N_tm_	2200	*μ*M	[123]
Free pyridine nucleotides concentration in cytoplasm	N_tc_	2000	*μ*M	Ad

Ul, unitless, Ad -Adjusted to fit glucose consumption rate and others conditions (see text).

**Table 3:** Standard parameter values (see text for explanations)

Model parameters:	Value	Units	Eq.	References
V_mglu_	0.011	*μ*M ms^-1^	1	Rescaled from Refs. [29,137]
K_mATP_	500	*μ*M	1	[26]
K_mgl_	7	mM	1	[26]
hgl	1.7	Ul	1	[26]
V_mGPD_	0.5	*μ*M ms^-1^	2	Ad
K_mG3P_	200	*μ*M	2	[138]
K_gNc_	0.09	Ul	2	[139]
V_mLD_	1.2	*μ*M ms^-1^	3	Ad
K_mLD_	47.5	*μ*M	3	[139]
k_LNc_	1	Ul	3	[139]
V_mPDH_	0.3	*μ*M ms^-1^	4	[138]
K_mpyr_	47.5	pM	4A	[139]
K_PNm_	81	Ul	4B	[139]
U_1_	1.5	Ul	4C	[29]
U_2_	1.1	Ul	4C	[29]
K_Cam_	0.05	*μ*M	4C	[29]
v_me_	22	*μ*M ms^-1^	5	Ad
K_mNH_	3000	*μ*M	5A	[123]
k_AT_	-0.00492	mV^-1^	5C	Fit to [24]
k_BT_	-0.00443	mV^-1^	5C	Fit to [24]
V_mph_	8	*μ*M ms^-1^	7	Ad
K_mADP_	20	*μ*M	7A	[26]
hph	2	Ul	7A	[26]
hp	8	Ul	7B	Ad
K_Ph_	131.4	mV	7B	Ad
K_PCam_	0.165	*μ*M	7C	[130]
P_lb_	0.0012	*μ*M ms^-1^	8	Ad
P_lr_	0.0012	*μ*M ms^-1^	8	Ad
k_lp_	0.0305	mV^-1^	8	Fit to [24]
T_NADH_	0.05	*μ*M ms^-1^	9	Ad
K_TNm_	16.78	Ul	9	[76]
K_TNc_	0.002	Ul	9	Ad
P_Ca_	0.004	ms^-1^	10	Ad
a_m_	0.2	Ul	10	[130]
a_i_	0.341	Ul	10	[130]
V_mNC_	0.025	*μ*M ms^-1^	11	Ad
K_Naj_	8000	*μ*M	11	[130]
K_Caj_	8	*μ*M	11	[130]
k_gpd_	0.00001	ms^-1^	12	Ad
k_NADHm_	0.0001	ms^-1^	14	Ad
k_NADPc_	0.0001	ms^-1^	16	Ad
*k*_ATP_	0.00004	ms^-1^	20	Ad
*k*_ATP,Ca_	0.00009	*μ*M^-1^ms^-1^	20	Ad
[Ca^2+^]_R_	0.09	*μ*M	23	Ad
k_ACa_	0.25	*μ*M	23	Ad
K_AD_	25	Ul	23	Ad
hCa	4	Ul	23	Ad

Ad -Adjusted to fit glucose consumption rate and others conditions (see text).

#### Glucose uptake and glycolysis

When the concentration of glucose rises in the extracellular medium, the *β*-cells take up glucose by means of a glucose transporter, GLUT2 and/or GLUT1. The uptake is rapid and believed to effectively equilibrate the extracellular and intracellular concentrations of the sugar [6]. The initial phosphorylation of glucose to glucose 6-phosphate (G6P) is catalyzed by glucokinase (see Introduction).

In the absence of externally added pyruvate or other metabolites, *β*-cells are glycolytic. For the breakdown of each G6P molecule, the overall reaction yielded two pyruvate molecules, the phosphorylation of three ADP molecules to form ATP and the reduction of two NAD^+ ^molecules to NADH. Glucose phosphorylation is the key limiting step for the steady-state rate of glycolysis in *β*-cells. However, the overall rate of ATP synthesis at high workloads can also be limited by glycolysis due to a decreased cytoplasmic NAD^+ ^availability for glyceraldehyde 3-phosphate dehydrogenase [18,118].

We simulated the phosphorylation of glucose and glycolytic flux by equations for the limiting enzymes. The glucokinase reaction was modeled as previously [26]:(1)

where *J*_glu _is the rate of the glucokinase reaction, V_mglu _is the maximum rate of glucose consumption, [Glu] is extracellular D-glucose concentration, [ATP]_c _is the cytoplasmic ATP concentration, K_mATP _is the Michaelis-Menten constant, K_mgl _is the glucose concentration at which the reaction is half maximum, and hgl is the Hill coefficient.

We lumped several glycolytic reactions and simulated only the NAD^+^-dependent step. The flux through glyceraldehydes 3-phosphate dehydrogenase (*J*_gpd_) was assumed to follow irreversible Michaelis-Menten kinetics, where a dependence on [NAD^+^]_c_/[NADH]_c _was written as suggested previously [119]:(2)

where V_mGPD _is the maximal glyceraldehyde3-phosphate dehydrogenase activity, [G3P] is the cytoplasmic glyceraldehydes 3-phosphate concentration, K_mGPD _is the Michaelis-Menten constant, [NAD^+^]_c _and [NADH]_c _are the cytoplasmic NAD^+ ^and NADH concentrations, and K_GNc _is the [NAD^+^]_c_/[NADH]_c _ratio that gives half maximal glycolytic flux and NADH production.

#### Pyruvate reduction

The flux catalyzed by lactate dehydrogenase (*J*_LDH_) was described [119]:(3)

where V_mLDH _is the maximal lactate dehydrogenase activity, [Pyr] is the cytoplasmic pyruvate concentration, K_mLD _is the Michaelis-Menten constant, K_LNC _is the [NADH]_c_/[NAD^+^]_c _ratio that gives half lactate and NAD^+ ^production. Basal level of V_mLDH _was evaluated (Table 3) on the basis of the proposal that the rate of lactate output is approximately 5% of the rate of glucose consumption at [Glu] = 8 mM and other coefficients were at their basal level.

#### Pyruvate dehydrogenase

The cytoplasmic pyruvate concentration is the key factor for mitochondrial ATP synthesis. After being transported into the matrix of mitochondria, pyruvate is decarboxylated in a process catalyzed by the multienzyme complex pyruvate dehydrogenase (PDH), thus forming the intermediate acetyl-coenzyme, or carboxylated by pyruvate carboxylase to form oxaloacetate (anaplerotic pathway) [120,121]. As in a previous model [30] we assumed that the citric acid dehydrogenase rate is proportional to the reaction rate of the PDH reaction. We assigned PDH a commanding role in the regulation of flux of glycolytic metabolites into the TCA cycle. In this step NAD^+ ^is also reduced to NADH, and PDH activity is regulated by the availability of its coenzyme NAD^+^, i.e. its activity is decreased when high ratios of NADH/NAD^+ ^prevail [119]. Calcium also activates pyruvate dehydrogenase [80]. We described the rate of pyruvate consumption in mitochondria (*J*_PYR_) as the product of several regulated factors:(4)

where(4A)(4B)(4C)

V_mPDH _represents the maximal PDH activity, f_PYR _is the pyruvate kinetic factor, [NAD^+^]_m _and [NADH]_m _are the mitochondrial NAD^+ ^and NADH concentrations, f_PNAD _is the [NAD^+^]_m_/[NADH]_m _kinetic factors and F_PCa _is the calcium activity factor, and [Ca ^+^]_m _is the mitochondrial Ca ^+ ^concentration.

Equations for f_PYR _and f_PNAD _are based on the Equation 18 from [119], K_MPYR _is a phenomenological Michaelis-Menten constant, K_PNm _is the ([NAD^+^]_m_/[NADH]_m _ratio that gives half maximal NADH production. Equation 18 from [29] was used for F_PCa_, where u_1_, u_2 _and K_Cam _are the parameters for fraction of activated PDH.

#### Electron transfer chain (ETC)

Ψ_m _is maintained primarily by the action of respiration-driven proton pumps in the electron transport chain that use the energy contained in NADH and FADH2 to pump hydrogen ions (H^+^) across the mitochondrial membrane out of the mitochondrial matrix. This process depends on NADH, voltage and oxygen concentration.

As protons are pumped across the inner mitochondrial membrane oxygen is consumed by the ETC. Thus, the respiration-driven proton flux is linked to O_2 _consumption. Both NADH and FADH2 are electron donors, but in our model NADH is the primary and dominant donor. Therefore we were able to omit a contribution from FADH2. We described respiration in terms of an effective-driven proton flux. We based the equation on the conceptual model [56] where the steady-state flux of electrons through the ETC was represented as a product of several factors:(5)

where(5A)(5B)(5C)

*J*_hres _is the rate of proton pumping through ETC, V_me _is the rate at optimal conditions. F_De _is the donor kinetic factor, f_Ae _is the acceptor kinetic factors and, F_Te _is the thermodynamic potential factor.

f_De _was taken from the model [122]. Note that the apparent affinity of complex 1 for NADH (K_mNH _in Equation 5A) is very low for *in vitro *measurements and does not correspond to the measured affinity *in vivo *[34]. For this reason, we used K_mNH _= 3 mM (Table 3) that corresponds to the value found for this reaction *in vivo *[123].

Acceptor kinetic factor (f_Ae_) reflects the decrease in electron transport resulting from diminished oxygen availability. In our model oxygen concentration is assumed to be saturated and unlimited.

Thermodynamic potential factor (F_Te_) determines an inhibition of electron transport with an increase in Ψ_m_. This dependence was evaluated according to [24] where k_AT _and k_BT _are coefficients that were fitted mathematically for mitochondria from muscle cells.

Cellular oxygen consumption rate is an indicator of mitochondrial electron transport. Respiration rate was determined as O_2 _consumption (*J*_O2_). Then the oxygen consumption rate (*J*_O2_) can be calculated as(6)

The factor of 0.1 indicates that the full chain from NADH to oxygen is believed to translocate ten protons per oxygen atom consumed where the H^+^/O ratios for oxidation processes were assumed to be constant [124].

#### F_1_F_0 _ATPase

In an intact cell, under physiological high K^+^, a contribution of ΔpH to the protonmotive force is usually small [125,126]. For simplicity, we suggest that the F_1_F_0 _ATPase uses primarily the mitochondrial membrane potential to generate ATP from ADP and P_i _by allowing H^+ ^ions to flow into the mitochondria. Some experimental evidence of this has been reported [127]. The rate of proton flux through mitochondrial F_1_F_0 _ATPase (*J*_*ph*_) was also described as the product of the several specific factors:(7)

where(7A)(7B)(7C)

V_mph _is the rate of the proton flux under optimal conditions. A_D _is the kinetic factor for free cytoplasmic ADP, where [MgADP_f_]_c _is the concentration of free cytoplasmic MgADP, K_mADP _is the activation rate constant, and hph is the Hill coefficient. A_T _is the thermodynamic potential factor, where K_ph _is the membrane potential yielding half maximal ATP production, hp is the Hill coefficient. A_Ca _is a sum of kinetic factors describing the effect of calcium concentration, where K_PCam _is the [Ca ^+^]_m _binding constant.

Since ADP concentration in mitochondria cannot be readily measured in living cells, we used the dependence of A_D _on free MgADP in medium that can be calculated using the Hill equation, where the mitochondrial oxidative phosphorylation rate increases with increased free MgADP concentration. The apparent half-saturated concentration is in the range of 20 - 45 *μ*M and the Hill coefficient is about 2 [128,129]. Note that a supply of ADP from cytoplasm is provided by the action of adenine nucleotide translocator (ANT) on the mitochondrial inner membrane. We did not model this translocator because Equation 7A includes the kinetic properties of ANT [128].

**Figure 12:**
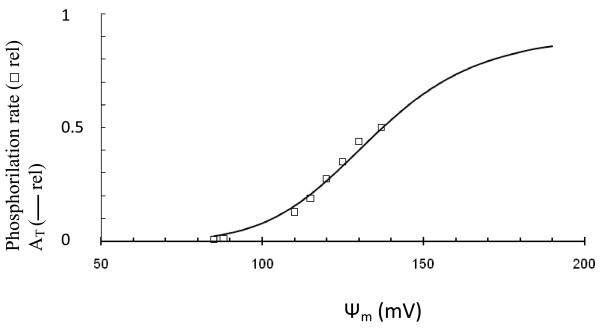
**Relative steady-state ATP production activity with respect to Ψ_m_**. Relative rate of phosphorylation (□) was based on experimental data [131] for human cell line mitochondria (their Figure 2B, relative average values) and model fit to Equation 7B for A_T _(solid line).

The thermodynamic potential factor (A_T_) can be represented as a sigmoid dependence of ATP production on mitochondrial membrane potential [130]. However, in contrast to [130] we used the data obtained by [131] for mammalian mitochondria to fit the coefficients for this dependence (Figure 12). The solid line shows the model fit where K_mp _is 131.4 mV and Hill coefficient is 8. A_T _voltage dependence saturates when Ψ_m _is above 160 mV. This dependence of ATP production on Ψ_m _was similar for mitochondria from muscle and *β*-cell [24]. Similar dependence of phosphorylation on Ψ_m _was found for rat liver mitochondria [132], and that is consistent with experimental findings for ox-heart submitochondrial vesicles [133]. A_Ca _is the again the sum of kinetic factors describing the effect of Ca^2+ ^[130]. Note that *J*_ph _increases with the electrical gradient (Ψ_m_), the MgADP concentration in cytosol and with the Ca^2+^concentration in mitochondria.

#### Proton leak

The mitochondrial membrane clearly leaks protons, decreasing the energy that can be used to drive ATP synthesis. Up to 20% of the basal metabolic rate may be dissipated in this basal leak, always present in mitochondria [124]. Part of the leak is also due to uncoupling proteins (UCP_s_) that exist in mitochondria to uncouple oxidative phosphorylation, among other possible functions, and the level of their expression varies. To simulate the effect of UCPs we used a special regulated proton leak coefficient. The dependence of the rate of proton leak back into the inner mitochondrial matrix on Ψ_m _was described as follows [24]:(8)

*J*_hl _is the proton leak across the mitochondrial inner membrane, P_lb _is the basal leak coefficient, and P_lr _is the regulated leak coefficient, k_lp _is the membrane potential coefficient.

We evaluated the basal leak coefficient in our model by supposing that the basal leak approaches ~20% of the electron transport rate at 160 mV (see Table 3). We set the equation so that the coefficient P_lr _equals P_lb _for basal *β*-cell simulation (Table 3), leading to a 2-fold increase of *J*_h1 _(i.e up to 40% of the electron transport rate at 160 mV).

#### NADH shuttles

Transport of the reducing equivalents derived from glycolysis in *β*-cells from the cytosol to mitochondria in exchange for NAD^+ ^is primarily through the glycerol phosphate and the malate-aspartate shuttles [2,17]. These shuttles involve oxidation and reduction reactions and include the enzymatic transporters. However, these processes have not been sufficiently described to be included in a model. Therefore, we employed an empirically derived rate expression [76], where the shuttle activity is expressed as the function of cytoplasmic and mitochondrial NADH/NAD^+ ^ratios:(9)

where T_NADH _is the transport rate coefficient, K_TNc _and K_TNm _are the affinity coefficients.

#### Ca^2+ ^uniporter for uptake of Ca^2+ ^into mitochondria (*J*_uni_)

Ca^2+ ^influx into the mitochondria is mediated by the Ca^2+ ^uniporter that is regulated by the electrochemical gradient. This Ca^2+ ^uniporter is not likely to be saturable by Ca^2+ ^under physiological conditions and exhibits a half-activation constant for cytoplasmic Ca^2+ ^at concentrations greater than 10 *μ*M [9]. The mitochondrial Ca^2+ ^uniporter was described according to [130] as(10)

where *P*_Ca _is the permeability of the Ca^2+ ^uniporter, Z_Ca _is the charge of Ca^2+^, [Ca^2+^]_c _is the cytoplasmic Ca^2+ ^concentration, *α*_m_, and *α*_i _are the mitochondrial and extramitochondrial activity coefficients.

#### Na^+^/Ca^2+ ^exchanger for release of mitochondrial Ca^2+ ^(*J*_NCa_)

In most cells, including pancreatic *β*-cells, the main mechanism of Ca^2+ ^extrusion from the mitochondria is the Na^+^/Ca^2+ ^exchanger [9,82]. The expression for the Na^+^/Ca^2+ ^exchanger was described according to [130] as(11)

where

*J*_NCa _is the Na^+ ^- Ca^2+ ^exchange flux, V_mNc _is the maximal exchanger velocity, [Na^+^]_c _and [Na^+^]_m _are the cytoplasmic and mitochondrial Na^+ ^concentration, k_Naj _is the binding constant for sodium and K_Caj _is the binding constant for Ca^2+^.

We set Ca^2+ ^uniporter and Na^+^/Ca^2+ ^exchanger maximum activity from experimental data obtained for living cells. Delay was observed between changes in the cytosolic Ca ^+ ^concentration and corresponding changes in [Ca^2+^]_m _[50,109]. This lag may reflect a delayed response of the [Ca^2+^]_m _on [Ca^2+^]_c _changes. We have modeled this delay by setting the maximal activities of the Ca^2+ ^uniporter and the Na^+^/Ca^2+ ^exchanger at levels that replicate experimental slow [Ca^2+^]_m _dynamics (see text).

#### Dynamic equations

Finally, based on the above relations we determined the dynamic equations for cytoplasmic [G3P], [Pyr], [NADH]_c _and free [ADP] and the mitochondrial variables: [NADH]_m_, [Ca ^+^]_m _and Ψ_m_*- *All fluxes were expressed as mass fluxes per unit time per unit total cell volume.

The balance equation for the G3P concentration is(12)

where k_gpd _is the rate constant of G3P consumption in cytoplasm, V_i _is the relative cytoplasmic fraction of total cell volume. Coefficient two in the numerator indicates that the breakdown of each glucose molecule yields two G3P molecules.

Pyruvate is the main product of glycolysis. Due to lack of information on pyruvate concentrations at the sub-cellular level in *β*-cells, we do not differentiate the cytosolic and mitochondrial pyruvate pools in this model. For simplicity, we assume that mitochondrial pyruvate is decarboxylated only in a process catalyzed by PDH. The equation describing [Pyr] change over time is(13)

where V_mmit _is the relative mitochondrial matrix fraction of the cell volume.

In this model the TCA cycle is not explicitly modeled. However, it is known that under steady-state conditions, four NADH and one FADH2 are synthesized in the TCA cycle for each pyruvate molecule. To help simplify the mitochondrial variables in our model, we evaluated the reducing equivalents (NADH and FADH2) in terms of H^+ ^flux due to pumping in the ETC. We assumed that 10 and 6 protons are pumped by each NADH and FADH2 oxidation, respectively [124]. This means that one FADH2 can be represented as 0.6 NADH molecule.

We assumed that the NADH production rate in mitochondria is determined by the reaction rate of pyruvate decarboxylation. The mitochondrial NADH concentration is decreased by the action of the ETC, during which NADH is converted to NAD^+ ^and oxygen is consumed. The following equation describes the change in [NADH]_m _over time(14)

where the stoichiometric coefficient 4.6 in the numerator arises from the number of NADH molecules that are produced from each pyruvate molecule. The coefficient 0.1 indicates that 10 protons are transported by the ETC for each NADH consumed [124]. k_NADHm _is the rate constant of NADH consumption in mitochondria. The concentration of pyridine nucleotides is assumed to be conserved in the mitochondrial matrix:(15)

where N_tm _is the total concentration of pyridine nucleotides in the mitochondrial matrix (Table 2).

We assume also that the cytoplasmic concentration of pyridine nucleotides is conserved. The balance equation for the cytoplasmic NADH and NAD^+ ^concentrations is(16)(17)

where N_tc _is the total concentrations of pyridine nucleotides in cytoplasm (Table 2). k_NADHc _is the rate constant of NADH consumption in the cytoplasm.

Mitochondrial Ψ_m _depends on the respiration module that establishes Ψ_m_, negative inside, and the dissipation processes: the activity of the phosphorylation apparatus, which includes the phosphate carrier, the ATP synthase and the adenine nucleotide translocase and proton-leak reactions. Another portion of Ψ_m _drives Ca^2+ ^out of the matrix via the Na^+^/Ca^2+ ^exchanger. Ca^2+^influx into mitochondria mediated by the Ca^2+ ^uniporter is also electrogenic. The final equation for Ψ_m _is(18)

where C_mi _is the mitochondrial membrane capacitance. The stoichiometric coefficient 2 in the numerator arises from the number of the charges that flow into mitochondria with one Ca^2+^. We assumed that Ψ_m _> 0.

The balance equations for [Ca^2+^]_m _is described by(19)

where f_m _is the constant describing the fraction of free Ca^2+ ^in mitochondria.

Several assumptions were made to simplify the ATP regulation model: (1) Due to the rapid export of mitochondrial ATP to the cytosol via the ATP/ADP transporter no limitation for this process was suggested. (2) ADP binding is in equilibrium (i.e. the binding reactions are fast compared to the other processes included in the model of ATP dynamic).

Utilization of ATP in the *β*-cells is mostly for ion transport, biosynthesis, and secretion. The rate of ATP utilization is a complex function of the concentrations of ATP, [Ca^2+^]_c_, glucose and numerous other factors, but almost nothing is known about the quantitative relation between them. Clearly, there is some basal level of ATP consumption in a cell at low glucose and [Ca^2+^]_c _concentrations. There is evidence that ATP hydrolyzing processes are accelerated during a glucose-induced increase in [Ca^2+^]_c _in *β*-cells, for example, ATP consumption can be increased due to increased Ca^2+ ^pump activity in the plasma membrane and endoplasmic reticulum [7,10,12,13]. For this reason, two terms for ATP consumption were introduced: basal and Ca^2+^-dependent (see [5]). Then, on the basis of the Equation 7 for oxidative phosphorylation we can write the balance equation for [ATP]_c _and [ADP]_c_(20)

where(21)(22)

The factor of 2 in the numerator of Equation 20 (at *J*_glu_) indicates that two ATP molecules are produced during glycolysis from each glucose molecule. The coefficient 0.231 (at *J*_pf_) arises from the ATP/H^+ ^coupling stoichiometry of 3/13 in mammalian mitochondria. This suggested as well the additional transport of one proton from the cytoplasm to the matrix that is associated with the movement of phosphate through the mitochondrial membrane [124]; *K*_ATP _is the permanent rate constant of basal ATP consumption, and *K*_ATP__ca_ is the rate constant of ATP consumption that accelerates as Ca^2+ ^increases.

The general concentration of intracellular nucleotides (A_0_) was assumed to be constant. The constants K_ATP _and K_ATP,Ca _(Equation 20) were fitted to simulate both the low and high glucose modeled rate of ATP production and ATP/ADP ratio in *β*-cells (see [5,26]) (Table 3). The coefficient 0.055 in Equation 22 was calculated as in our previous model [5,26].

#### External parameters

The mechanisms of [Ca^2+^]_c _and [Na^+^]_c _regulation and their interrelationships with other metabolic and ionic fluxes are incompletely understood. For this reason in our model we used their empirical determination as the external parameters, even though we previously developed a mathematical model for [Ca^2+^]_c _and [Na ]_c _regulation in *β*-cells [5,26]. [Na^+^]_c _was proposed as constant in this model (Table 2).

The relationship between [Ca^2+^]_c _and glucose at steady-state was calculated using the change of ATP/ADP ratio as an intermediate agent by an empirical Hill-type equation.(23)

where [Ca^2+^]_R _is the constant [Ca^2+^] for resting low glucose, k_ACa _is the coefficients, K_AD _is the [ATP]_c_/[ADP]_c _ratio that gives half maximal [Ca^2+^]_c_, hCa is the Hill coefficient. The parameters of Equation 23 were set to a value (Table 3) that produced a reasonable amplification of [Ca^2+^]_c _with increased glucose (see Results and Discussion).

### Model for independent cytoplasmic Ca^2+ ^oscillations

We used a simple mathematical model that creates a periodically varied independent [Ca^2+^]_c _change in cytoplasm to simulate the oscillations of the mitochondrial parameters in *β*-cells. The model described in this section is based on a simple model [134] and used only for simulation of independent Ca^2+ ^oscillations in the cytoplasm of a mean individual cell.

#### Ca^2+ ^current (I_VCa_)

where(25)(26)

g_mVCa _is the maximum conductance for *I*_VCa _(g_mVCa _= 20 *μ*S), V_P _is the plasma membrane potential.

#### Ca^2+^-activated K^+ ^current (I_KCa_)

where(28)

where g_mKCa _is the maximum conductance for *I*_KCa_, K_KCa _is the half-maximum Ca^2+ ^binding constant for *I*_KCa _(g_mKCa _= 25 *μ*S, K_KCa _= 0.25 *μ*M).

The differential equation describing time-dependent changes in the plasma membrane potential (V_p_) is the current balance equation:(29)

where C_m _is the cell membrane capacitance (C_mp _= 6158 fF [5])

The equations for [Ca^2+^]_c _dynamics can be written as:(30)

where f_i _is the fraction of free Ca^2+ ^in cytoplasm, F is Faraday's constant, V_ci _is the volume of the cytosolic compartment in single cell (0.764 pL [5]), and *k*_sg _is the coefficient of the sequestration rate of [Ca^2+^]_c _(*k*_sg _= 0.00002 ms^-1 ^[5]).

### Computational aspects

For computational purposes we considered the *β*-cell as an assemblage of mitochondria with similar properties. The units used in the model are time in millisecond (ms), voltage in millivolts (mV), concentration in micromoles/liter (*μ*, M), flux in *μ*mol ms^-1^.

A factor (0.31) was used to convert picomoles per islet from metabolism experiments to the cytoplasmic millimolar terms of a single *β*-cell (as calculated in Ref. [29]). The mitochondrial protein density for total mitochondria volume is estimated at ~0.3 mg protein/*μ*l, and free water volume in mitochondrial matrix space can be estimated to be 0.24 of total mitochondria volume [135]. In line with these data we used the factor 1.25 to convert the measured nanomoles per milligram mitochondrial protein (nmol mg^-1 ^protein) to mitochondrial matrix space in millimollar terms. In our model the fluxes were specified for the volume of a cell. Multiplying the mitochondrial matrix volume (Table 2) by its protein density (1.25 g protein ml^-1^) by unit flux [in nmol (mg protein min^-1^)] gives the total flux in the mitochondria matrix per unit cell volume.

The general concentration of intramitochondrial and cytoplasmic adenine and purine nucleotides were kept constant during simulations (Table 2). Model parameters were found by several methods. Specifically, they were obtained from the scientific literature when possible and were also found by fitting specific model equations to experimental data. The third method was to estimate the parameter so that model variable values and time courses closely matched experimental data. Several enzymatic activity values were treated as adjustable parameters, which were adjusted using the reaction stoichiometries to reflect the rate of glucose phosphorylation by glucokinase (Table 3). Parameter values from Tables 2 and 3 were used unless otherwise mentioned. Nine ordinary differential equations (Equations 12-14, 16, 18-20, 29, 30) describe the behavior of [G3P], [Pyr], [NADH]_m_, [NADH]_c_, Ψ_m_, [Ca^2+^]_m_, [ATP]_c_, V_p _and [Ca^2+^]_c_. Coefficients are shown in Tables 2 and 3. Calculations on Figure 12 and a generation of all figures were performed using "Igor" (IGOR Pro, WaveMetrics, Inc, Lake Oswedo, OR, USA) and Microsoft Excel X.

Simulations were performed as noted previously for an idealized mean individual cell using the software environment from "Virtual Cell" (Fridlyand et al. [5,26]). To calculate the steady-state cellular parameters, the model was allowed to run for at least 10s with no external stimulation. Calculations obtained with the coefficients from Tables 2 and 3 have been mentioned in the text as a simulation at basal levels.

This model is available for direct simulation on the website "Virtual Cell" http://www.nrcam.uchc.edu in "MathModel Database" on the "math workspace" in the library "Fridlyand" with the name "GlucoseSensitivity-1" for the general model and with name "GlucoseSensitivity-2" for the general model that also includes independent [Ca^2+^]_c _oscillations.

## List of abbreviations

ATP/ADP: ratio of ATP to ADP; ETC: electron transport chain; EtBr: ethidium bromide; GK: glucokinase; Glu: glucose; GSIS: glucose-stimulated insulin secretion; G3P: glyceraldehyde 3-phosphate; K_ATP_: ATP-sensitive K^+ ^channels; LDH: lactate dehydrogenase; PDH: pyruvate dehydrogenase; PYR: pyruvate; ROS: reactive oxygen species; TCA: tricarboxylic acid; Tfam: mitochondrial transcription factor A; UCP2: uncoupling protein 2; Ψ_m_: mitochondrial membrane potential; **Subscript **c: cytoplasmic compartment; m: mitochondrial compartment.

## Competing interests

The authors declare that they have no competing interests.

## Authors' contributions

All authors (LF, LP) contributed equally to the formulation of the model, the estimation of parameters, the biological interpretations and conclusions, and the writing and editing of the manuscript. LF performed construction and simulation of the initial mathematical model. Both authors read and approved the final manuscript.
